# Quality assessment of a training program for undergraduate sonography peer tutors: paving the future way for peer-assisted learning in medical ultrasound education

**DOI:** 10.3389/fmed.2025.1492596

**Published:** 2025-03-03

**Authors:** Johannes Weimer, Didem Yilmaz, Carlotta Ille, Julia Weinmann-Menke, Lukas Müller, Hans Büchner, Holger Buggenhagen, Marie Stäuber, Ricarda Neubauer, Lone Winter, Andreas Weimer, Klaus Dirks, Julian Künzel, Maximilian Rink, Anna Dionysopoulou, Liv Lorenz, Roman Kloeckner, Florian Recker, Lina Schiestl

**Affiliations:** ^1^Rudolf Frey Learning Clinic, University Medical Center of the Johannes Gutenberg University Mainz, Mainz, Germany; ^2^I. Department of Medicine, University Medical Center of the Johannes Gutenberg University Mainz, Mainz, Germany; ^3^Department of Diagnostic and Interventional Radiology, University Medical Center of the Johannes Gutenberg University Mainz, Mainz, Germany; ^4^Department of Obstetrics and Prenatal Medicine, University Hospital Bonn, Bonn, Germany; ^5^Center of Orthopedics, Trauma Surgery, and Spinal Cord Injury, Heidelberg University Hospital Heidelberg, Heidelberg, Germany; ^6^Medical Clinic II, University Hospital Würzburg, Würzburg, Germany; ^7^Department of Otorhinolaryngology, Head and Neck Surgery, University Hospital Regensburg, Regensburg, Germany; ^8^Department of Gynecology and Obstetrics, University Medical Center of the Johannes Gutenberg University Mainz, Mainz, Germany; ^9^Department of Radiation Oncology and Radiotherapy, University Medical Center of the Johannes Gutenberg University Mainz, Mainz, Germany; ^10^Institute of Interventional Radiology, University Hospital Schleswig-Holstein - Campus Lübeck, Lübeck, Germany

**Keywords:** peer-assisted learning, peer-tutoring, undergraduate ultrasound education, peer tutors, sonography curriculum

## Abstract

**Introduction:**

Peer tutoring has been increasingly used to support university sonography teaching, necessitating well-qualified tutors. This study aims to evaluate the quality of a training program for sonography peer tutors developed and implemented at a university hospital.

**Materials and methods:**

A training program consisting of 11 modules was developed and subsequently evaluated for success with two subjective and two objective assessments of peer-tutoring quality. The two subjective assessments measured subjective scores of the peer tutors’ specialist and didactic skills using a Likert scale (1 = very low; 7 = very high) from the perspective of the individuals the peer tutor taught (assessment 1) and from the peer tutors themselves (assessment 2). The peer tutors also rated the training concept itself. The objective assessments evaluated the peer tutors’ specialist skills with a theoretical test (assessment 3) and a practical examination (assessment 4). Data collection for assessment 1 began in 2017, while data for assessments 2 to 4 were collected from 2021 to 2024.

**Results:**

A total of 2,980 data sets for assessment 1, 92 data sets for 2, 44 data sets for assessment 3, and 147 data sets for assessment 4 were included in the analysis. Peer tutors scored highly positively on assessments 1 [6.6 ± 0.63 scale points (SP)] and 2 (5.53 ± 0.63 SP), and these results remained consistently high throughout the semesters. Assessments 3 (74.7 ± 7.9%) and 4 (85.6 ± 10.5%) also showed strongly positive values that remained constant over time. Assessment 1 results were significantly higher than the others (*p* < 0.01.), while no significant differences were found between assessments 2, 3, and 4.

**Conclusion:**

The data indicate that the training concept developed established and maintained high-quality peer-tutor training throughout the reviewed semesters. Future efforts should promote the development of national and international standards for peer-tutor training and provide certification opportunities for peer tutors.

## Introduction

### Background

Diagnostic ultrasound has become increasingly important in clinical medicine. With developments in ultrasound technology and its advantages over alternative imaging methods, sonography is now taught to a range of students (1, 2). From the very beginning of residency training, young doctors are expected to be able to assess ultrasound findings adequately (3, 4). Accordingly, practical and theoretical sonography skills are taught at university (5–10).

The clinical environments of university hospitals are often characterized by limited resources, time constraints, and insufficient manpower, making it challenging to allocate staff for student education, particularly for small-group instruction (11, 12). Ultrasound training, in particular, requires instruction in a broad range of skills, including theory, equipment handling, image acquisition, interpretation of findings, and communication. These demands present significant personnel and financial challenges to medical faculties (13). To address these challenges more effectively, training concepts supported by peer tutoring have been developed and established in recent years (8, 9, 14, 15). Peer tutors are non-professional instructors who belong to the same social group as the students and typically possess a comparable level of knowledge and experience (16–18).

The use of student peer tutors to support qualified instructors is well established in medical training, as it enables effective teaching with close supervision even when personnel resources are limited. Peer tutors can reduce group sizes, thereby increasing the time available for students to engage in practical exercises (8, 9). For example, peer tutors have been successfully employed as “dissection assistants” in anatomy teaching (17, 18). Similarly, peer tutors have also been used in sonography teaching, ensuring hands-on training in small groups (14, 19–25), a practice also recommended by international ultrasound societies (5, 9).

Two types of peer tutoring are widely documented. In peer-assisted learning (PAL), the tutor and student are at the same learning level, while in near-peer tutoring, the tutor and student are at different academic levels (26). High-quality training for peer tutors is an important cornerstone in ensuring the quality of PAL (13, 21, 27–29). Consequently, structured and sustainable training concepts are necessary to equip peer tutors with the appropriate practical and didactic skills.

### Research problem and aim

Although international specialist societies advocate using peer tutors for ultrasound teaching (5, 8, 9), no national or international standardized training curriculum exists for peer tutors. Peer-tutor training is usually only marginally discussed in currently published sonography curricula (19, 21, 30, 31). Few studies specifically address ultrasound peer-tutor training (13, 21, 27, 29, 32). The training of peer tutors often includes participation in ultrasound courses (21, 29, 32, 33), clinical clerkships or observerships (32, 34, 35), and didactic training (29, 36, 37). In the process participation in ultrasound courses primarily imparts theoretical knowledge and practical examination techniques on healthy volunteers, while clinical clerkships focus on patient examinations and expand the students’ understanding of pathology. This study aims to describe and evaluate a training concept for sonography peer tutors that was developed and implemented at a university hospital.

## Materials and methods

### Study design

**Figure 1:**
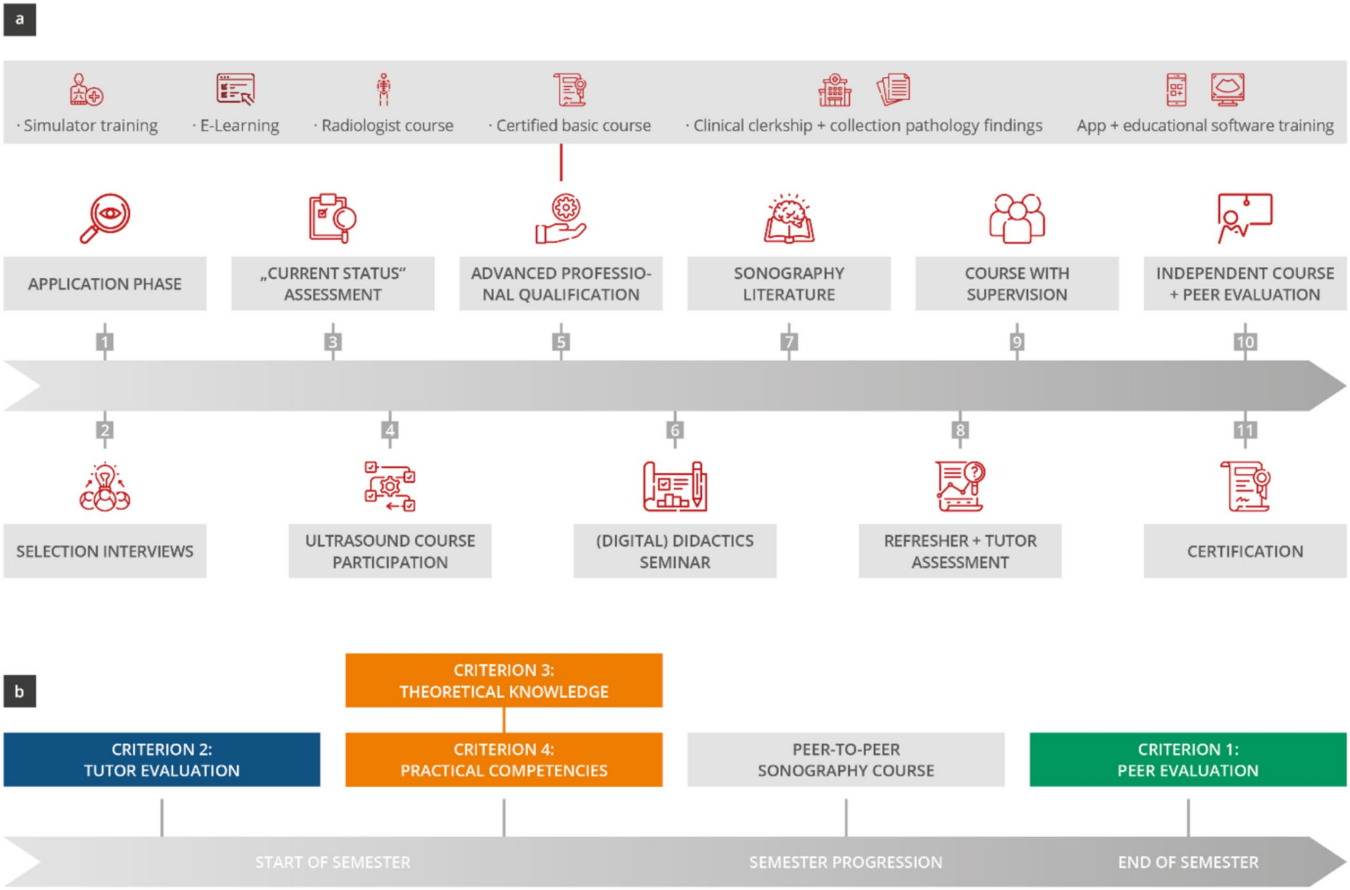
Presentation of the current Sonography peer tutor education curriculum. **(A)** The training curriculum consists of 11 modules that are to be completed within 1 year. **(B)** Regular theoretical and practical tests are conducted at the beginning of each semester as objective assessments, alongside evaluations conducted at both the beginning and end of each semester as subjective assessments. These measures are carried out to ensure the quality of the peer tutors who have already completed their training.

This prospective single-center observational study was conducted at a German university hospital (38). The developed “peer-tutor training curriculum for sonography education,” which consists of 11 modules, was evaluated using four assessments (two subjective and two objective) (Figure 1). The study included third-year undergraduate medical students enrolled in the peer-to-peer sonography course who completed the course evaluation, as well as trained peer tutors from various clinical semesters who fully participated in self-evaluations and theoretical and practical tests. Data collection took place over 14 semesters (Semester 1 to Semester 14) between 2017 and 2024.

### Sonography peer tutor education curriculum

The training curriculum for sonography peer tutors was developed and realized through interdisciplinary collaboration between students, didactic experts, and ultrasound specialists from various departments including internal medicine, otorhinolaryngology, radiology, and gynecology, and was based on current literature (5, 8, 27, 29, 36, 39, 40). Launched in 2017 and continuously refined until 2022, the training curriculum consisted of 11 modules completed by prospective tutors within approximately 1 year (see Figure 1). After completing the training, the tutors received time-based financial compensation for their work.

#### Module 1: application phase

The application phase served to recruit new potential peer tutors. Information and job advertisements were published during the last and penultimate pre-clinical semesters (and in the first and second clinical semesters) via printed posters, online posts, and information emails sent via the Student Office. The respective notices contain a clear profile of requirements (current semester, expectations of applicants, advantages of completing the program, duration of training, contact person, and information required). Applications received were then reviewed against the aspects listed in the requirement profile and the information provided by the applicants.

#### Module 2: selection interviews

Next, all applicants who met the required assessments were invited to an interview. Questions were asked about their previous experience, motivation for the role, and other relevant qualifications. This took place in small groups of three applicants and four or five current tutors (including a physician supervisor) using a predefined list of questions. A score was determined after each interview and the final selection of applicants was made once all interviews had been completed.

#### Module 3: assessment of status

After the selection process, all new tutors, current tutors, and the physician project manager met to assess the tutors’ current ultrasound skills and previous teaching experience. This module was used for self-reflection and to record the tutors’ practical expectations of the training, as well as to assess their motivation to teach the course content.

#### Module 4: course participation

The new tutors first completed an ultrasound course consisting of a basic workshop with 26 teaching units, in which the focus was on practical training with healthy volunteers as well as teaching theoretical content (41). The trainers were current tutors and former tutors, and the course was supervised by a physician. New tutors were given ultrasound lecture notes that were checked before by ultrasound experts. These notes contained all the important physical and technical basics of the specialty as well as standard sections and the most important pathologies. The new tutors also accessed a correspondingly developed e-learning course with videos (42).

#### Module 5: advanced professional qualification

This module formed the core of the theoretical and practical training for new tutors. It was divided into various sub-areas, some of which can be completed simultaneously. These included simulator training (10 units), the completion of an e-learning course (24 units), participation in a radiology course (20 units), a DEGUM-certified basic course (24 units), app-based training (20 units), and a clinical traineeship of 3–4 weeks requiring the collection of 100 findings.

#### Module 6: digital didactics seminar

This module provided didactic training for the new tutors. Participants were given lecture notes (Supplementary Table 1) and were shown training videos. The videos presented examples of “very good,” “good,” and “less good” reactions to teaching scenarios on the topics of “briefings,” “lesson introductions,” “practical instruction on equipment,” “picture explanations,” “use of teaching materials,” “structural labeling/naming,” “feedback,” “lesson conclusions,” and “Direct Observation of Procedural Skills (DOPS) implementation.” The students were asked to reflect on this learning. A workshop (24 units) then specifically addressed the above skills through a mix of theoretical units taught in plenary sessions and small group work led by didactic experts and medical alumni tutors.

#### Module 7: sonography literature

The new tutors were provided with a recommended list of current sonography publications selected by former tutors and physician supervisors. Independent study of the literature, designed to take place over semester vacations, was intended to deepen the new tutors’ technical and didactic knowledge and support the clinical training process.

#### Module 8: refresher + tutor assessment

Together with working tutors, the new tutors took part in a refresher workshop at the beginning of the semester. This workshop consolidated the practical and didactic skills taught previously and covered approximately 12 teaching units. During these refresher workshops, tutors were also presented with the latest training technologies and current innovations in relation to the course. During this workshop, new tutors underwent assessment 2 (“tutor evaluation”) and the theoretical and practical skills assessments 3 and 4 (“theory test” and “DOPS,” respectively) (43).

#### Module 9: course with supervision

All new tutors complete their first course under at least partial supervision of an experienced tutor acting as a mentor. The supervision involved regular class observations and feedback meetings. In addition, the new tutors could obtain feedback from the participants voluntarily at the end of a course lesson by completing a lesson evaluation form (Supplementary Table 2).

#### Module 10: independent teaching in a course and peer evaluation

Independently conducting an ultrasound course as a tutor was the primary goal of the training. Consultation with other tutors, former tutors, and physician supervisors was also possible at any time during this module. At the end of the course, course participants gave an overall evaluation of all tutors, including those already fully trained (assessment 1: “peer evaluation”).

#### Module 11: certification

After completing the entire training curriculum and conducting their course, tutors received a training certificate.

### Measuring the quality of the training curriculum

The quality of the training curriculum and the trained peer tutors was measured through four assessments (see Figure 1; Table 1) (43–45). These consisted of two subjective assessments (1 and 2) and two objective assessments (3 and 4). The quality of the training concept was assessed based on the average teaching skills demonstrated by the trained tutors each semester.

**Table 1:** Overview of the quality assessments for evaluating the developed sonography peer tutor training concept.

Content	Timepoint and way of collection	Methods
Assessment 1: peer evaluation (evaluation of tutoring skills by course participants)		
Evaluation of the tutors’ specific ultrasound (8 items) and didactic (6 items) skills by the peer group.	Online survey at the end of the semester.	Likert scale (1 = very bad; 7 = very good).
Assessment 2: tutor evaluation (self-assessment of skills and rating of the training concept)		
Tutors’ self-evaluation included baseline characteristics (7 items); satisfaction with the training undertaken (15 items); current skills in using teaching materials (11 items); didactic (9 items), specific ultrasound (8 items), and social (7 items) competencies; and motivations (10 items).	Online survey at the beginning of the semester.	Likert scale (1 = very bad; 7 = very good), dichotomous (“yes/no”) and open questions.
Assessment 3: theoretical knowledge (theoretical test*)		
Test (max. 90 points) covering the topics “Basics” (14 points), “Normal findings” (48 points), and “Pathologies” (28 points) of abdominal sonography*.	Digital assessment at the beginning of each semester.	Open questions
Assessment 4: practical competencies (practical test*)		
Examination procedures and pathologies of abdominal sonography (max. 49 points)*.	A written and standardized examination sheet at the beginning of each semester marked by a physician	1–2 DOPS tests

*Example questions and examination sheets are provided in Supplementary Tables 3, 4.

### Data collection and statistical methods

Data was collected using LimeSurvey (LimeSurvey GmbH, Germany) and written questionnaires. All data were saved in an Excel spreadsheet (Microsoft Excel^®^ Version 16.48, Microsoft Corporation, Redmond, WA, United States). All statistical analyses were performed in Rstudio (Rstudio Team [2020]. Rstudio: Integrated Development for R. Rstudio, PBC,^1^ last accessed on 20 042024) with R 4.0.3 (A Language and Environment for Statistical Computing, R Foundation for Statistical Computing,^2^ last accessed on 20 042024).

Where possible, a main scale score was derived from the average of the subscale scores. The internal consistency of the scales was tested and confirmed by calculating Cronbach’s alpha reliability. Binary and categorical baseline variables are given as absolute numbers and percentages. Continuous data are given as median and interquartile range (IQR) or as mean and standard deviation (SD). Categorical variables were compared using Chi squared test and continuous variables using the T-test or the Mann–Whitney U test. Additionally, parametric (ANOVA) or non-parametric (Kruskal-Wallis) analyses of variance were calculated and further explored with pairwise post-hoc tests (T-test or Mann–Whitney U). Tests of differences in the total scores for both subjective and objective measures were carried out for each semester and across semesters. For this purpose, all scores determined were converted into percentages from 0 to 100%, where 100% corresponds to the maximum (7 on the Likert scale or the highest possible score in the tests) and 0% corresponds to the minimum (1 on the Likert scale or the lowest possible score in the tests). *p*-values <0.05 were considered statistically significant.

## Results

### Data description

The reliability tests, according to Cronbach’s alpha, show that the internal consistency of the main scales, in a range of 0.83–0.92, did not vary considerably.

### Results of assessment 1: evaluation of tutoring skills by course participants (peer evaluation)

**Figure 2:**
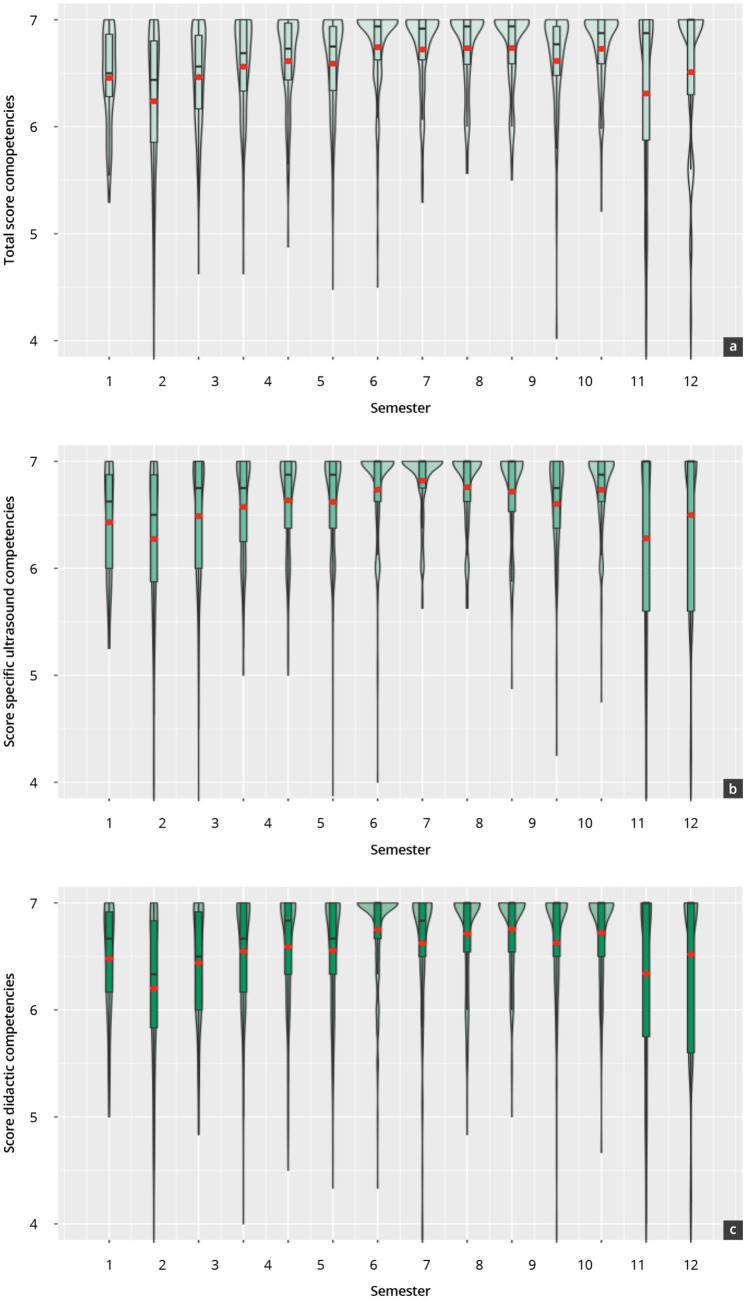
Evaluation results of tutor competencies by course participants from 2017 to 2014 across “total competencies” **(A)**; “specific ultrasound competencies” **(B)**; and “didactic competencies” **(C)**.

A total of N = 2,987 participant evaluations were included in the analysis over a total of 14 semesters between 2017–2024 (see Supplementary Table 5 and Figure 2). The general (6.58 ± 0.59), specific ultrasound (6.58 ± 0.63), and didactic competencies (6.57 ± 0.64) of the tutors were evaluated by the participants in high scale ranges [>6 scale points (SP)]. This is also true for the surveyed sub-items, which were evaluated in the constant range between 6 and 7 SP across the 14 semesters under review. No significant differences (*p* = 0.4) were found in the overall value between the evaluations of specific ultrasound and didactic skills, which also is the case for the individual semester evaluations.

### Results of assessment 2: tutor evaluation

A total of *N* = 92 tutor evaluations were included in the analysis from Semester 12 to Semester 14 (see Supplementary Table 6). The gender, semester, and age distribution of the tutors remained relatively consistent during this period. All tutors were in at least their 4th semester, had held an average of approximately 4 courses independently, and had performed more than 150 sonographies.

**Figure 3:**
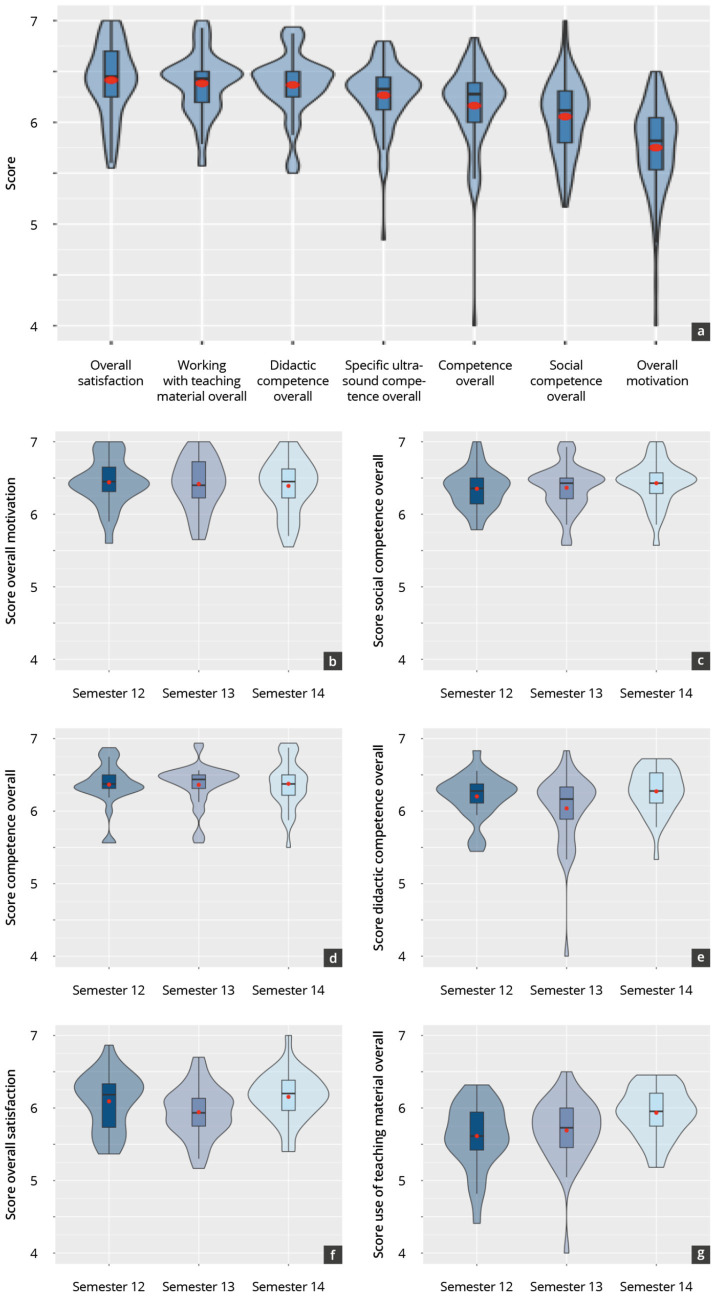
Tutor self-evaluation results across all semesters per item **(A)** and in total by semester **(B–G)**.

Figure 3 and Supplementary Table 7 show the results of the tutor self-evaluations for the main items. The ratings remained consistently similar within a similar scale range of 4.5–5.9 SP across the observed semesters. “Overall motivation” tended to receive the highest evaluations, while “working with the teaching material overall” received the lowest. However, within the latter category, significantly higher ratings were recorded across the semesters (*p* = 0.01).

**Figure 4:**
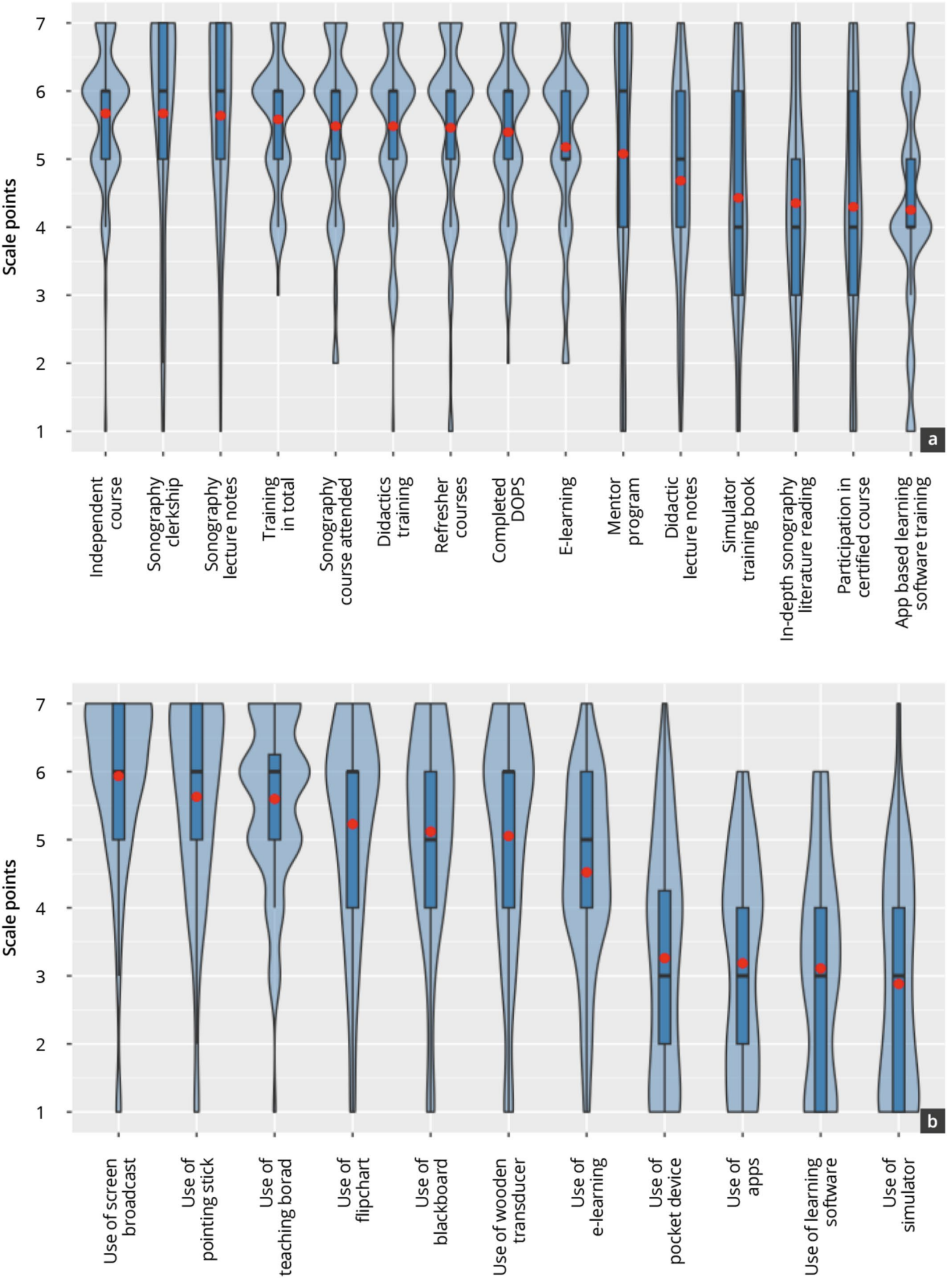
Evaluation results of the sub-items making up satisfaction with the tutor training concept **(A)** and skills in using the teaching materials **(B)** across all semesters.

Figure 4 and Table 2 show the results of the peer-tutor evaluation in reported satisfaction with the peer-tutor training concept, and their current skills in using teaching materials. Overall, satisfaction with the training concept remained constant moderate over the period from Semester 12 to Semester 14 (*p* = 0.06). The evaluation results of semester 13 tended to be slightly lower in the main and subitems than in the other semesters. In particular, the results of the evaluation of “didactics training” (*p* = 0.03), “sonography courses attended” (*p* = 0.02), “independent course” (*p* < 0.01), and “training in total” (*p* = 0.02) were significantly lower in semester 13 than in semester 14.

**Table 2:** Evaluation results of the main and subitems assessing peer-tutor satisfaction with the peer-tutor training concept and their ability to use the teaching materials from semesters 12 to 14.

Item	Overall	Semester 12	Semester 13	Semester 14	*p*-value
Satisfaction with the components of the training concept					
Satisfaction overall	5.11 ± 0.73	5.19 ± 0.77	4.89 ± 0.71	5.31 ± 0.67	0.06
Training overall	5.58 ± 0.93	5.6 ± 1.12	5.26 ± 0.85	5.94 ± 0.73	0.01
Didactics training	5.48 ± 1.21	5.16 ± 1.55	5.31 ± 0.99	5.94 ± 1.0	0.03
Sonography course	5.48 ± 1.29	5.48 ± 1.3	5.14 ± 1.29	5.87 ± 1.2	0.05
E-learning	5.18 ± 1.24	5.08 ± 1.35	5.2 ± 1.26	5.23 ± 1.18	0.93
Sonography lecture notes	5.64 ± 1.34	6.0 ± 1.0	5.54 ± 1.31	5.45 ± 1.57	0.39
Didactic lecture notes	4.68 ± 1.44	4.92 ± 1.32	4.31 ± 1.41	4.9 ± 1.54	0.12
Simulator training book	4.43 ± 1.54	4.6 ± 1.5	4.17 ± 1.67	4.58 ± 1.43	0.53
Participation in a certified course	4.3 ± 1.78	4.24 ± 1.76	4.17 ± 1.62	4.48 ± 1.96	0.69
Sonography clerkship	5.67 ± 1.57	5.68 ± 1.7	5.54 ± 1.8	5.81 ± 1.17	0.9
Mentor program	5.08 ± 1.92	5.04 ± 1.97	4.83 ± 1.93	5.39 ± 1.89	0.4
App-based training	4.25 ± 1.55	4.48 ± 1.42	3.8 ± 1.55	4.58 ± 1.59	0.11
In-depth sonography literature reading	4.35 ± 1.58	4.64 ± 1.6	4.23 ± 1.52	4.26 ± 1.65	0.44
Completed DOPS	5.4 ± 1.11	5.44 ± 1.16	5.2 ± 0.96	5.58 ± 1.23	0.27
Independent course	5.67 ± 1.02	5.84 ± 0.9	5.29 ± 1.18	5.97 ± 0.8	0.04
Refresher courses	5.46 ± 1.4	5.48 ± 1.76	5.29 ± 1.32	5.65 ± 1.17	0.36
Skills in the use and handling of teaching materials					
Overall use of teaching materials	4.5 ± 0.87	4.23 ± 0.92	4.38 ± 0.9	4.87 ± 0.68	0.01
Use of e-learning	4.52 ± 1.39	4.5 ± 1.61	4.46 ± 1.29	4.61 ± 1.36	0.84
Use of the pointing stick	5.63 ± 1.48	4.85 ± 1.74	5.69 ± 1.45	6.23 ± 0.92	0.003
Use of the teaching board	5.6 ± 1.214	5.23 ± 1.34	5.51 ± 1.27	6.0 ± 0.93	0.07
Use of apps	3.19 ± 1.55	2.96 ± 1.66	3.09 ± 1.34	3.48 ± 1.69	0.46
Use of the wooden transducer	5.05 ± 1.68	4.39 ± 1.84	4.97 ± 1.64	5.71 ± 1.37	0.008
Use of learning software	2.88 ± 1.63	2.85 ± 1.76	2.66 ± 1.37	3.16 ± 1.79	0.56
Use of the blackboard	5.12 ± 1.56	4.73 ± 1.71	5.09 ± 1.6	5.48 ± 1.34	0.24
Use of the flipchart	5.23 ± 1.53	5.0 ± 1.5	5.06 ± 1.73	5.61 ± 1.28	0.22
Use of the screen broadcast	5.94 ± 1.35	5.92 ± 1.52	5.83 ± 1.58	6.07 ± 0.85	0.89
Use of the simulator	3.11 ± 1.62	3.27 ± 1.56	2.8 ± 1.53	3.32 ± 1.76	0.43
Use of the pocket device	3.26 ± 1.7	2.81 ± 1.55	3.06 ± 1.59	3.87 ± 1.8	0.06

The subitems “app-based training,” “in-depth sonography literature reading,” “didactic lecture notes,” “simulator lecture notes training,” and “participation in a certified course” were evaluated on average in significantly lower scale point ranges (4.2–4.7 SP, *p* < 0.05) per semester than the other items (scale point ranges 5.1–5.7 SP).

The reported skills of using and handling the teaching materials tended to increase in the overall score over the period under consideration (*p* = 0.01). In particular, the “use of the pointing stick,” “use of the teaching board” and “use of screen broadcasts” were evaluated per semester in significantly higher scale point ranges (4.8–6.3 SP, p < 0.05) than the “use of the simulator,” “use of the pocket device“, “use of learning software” and “use of apps” (2.8–3.9 SP).

The tutor self-evaluation results of their “didactic,” “specific ultrasound,” and “social” competencies are presented in Table 3.

**Table 3:** Results of the peer-tutor self-evaluation of didactic, thematic, and social competence and motivation from semesters 12 to 14.

Item (Likert scale 1 = very low, 7 = very high)	Overall	Semester 12	Semester 13	Semester 14	*p*-value
Didactic competencies					
Overall didactic competencies	5.33 ± 0.82	5.41 ± 0.63	5.08 ± 1.01	5.55 ± 0.63	0.06
General didactic competence	5.26 ± 0.94	5.35 ± 0.75	5.03 ± 1.07	5.45 ± 0.89	0.22
Use of learning materials during course lesson	5.22 ± 0.97	5.23 ± 0.86	4.86 ± 1.12	5.61 ± 0.72	0.006
Topic presentation	5.36 ± 1.04	5.58 ± 0.9	5.03 ± 1.12	5.55 ± 1.0	0.04
Lesson introduction	5.4 ± 1.08	5.62 ± 0.98	5.06 ± 1.24	5.61 ± 0.88	0.05
Lesson design	5.29 ± 0.92	5.46 ± 0.81	4.97 ± 1.04	5.52 ± 0.77	0.04
Guidance of participants on the device	5.58 ± 0.99	5.69 ± 0.68	5.49 ± 1.15	5.58 ± 1.03	0.91
Transducer handling correction	5.44 ± 1.08	5.62 ± 0.85	5.26 ± 1.38	5.48 ± 0.85	0.76
Closing and summary	5.38 ± 1.14	5.23 ± 1.21	5.26 ± 1.25	5.65 ± 0.92	0.31
Carrying out tests	5.02 ± 1.34	4.89 ± 1.18	4.74 ± 1.63	5.45 ± 1.0	0.15
Thematic competencies					
Overall thematic competencies	5.74 ± 0.62	5.73 ± 0.62	5.73 ± 0.6	5.75 ± 0.67	0.98
Knowledge	5.41 ± 0.74	5.42 ± 0.7	5.43 ± 0.74	5.39 ± 0.8	0.98
Using oft the device	5.34 ± 0.95	5.31 ± 1.09	5.31 ± 0.76	5.39 ± 1.05	0.88
Transducer handling	5.89 ± 0.76	6.0 ± 0.75	5.8 ± 0.72	5.9 ± 0.83	0.71
Spatial orientation	6.03 ± 0.86	6.0 ± 0.94	6.0 ± 0.84	6.1 ± 0.83	0.91
Sono-anatomical correlation	5.89 ± 0.78	5.89 ± 0.82	5.86 ± 0.73	5.94 ± 0.81	0.88
Visualization of organs	5.65 ± 0.76	5.58 ± 0.76	5.69 ± 0.72	5.68 ± 0.83	0.84
Examination and assessment of the organs	5.85 ± 0.81	5.46 ± 0.86	5.8 ± 0.72	5.74 ± 0.86	0.38
Patient guidance	5.99 ± 0.86	6.19 ± 0.69	5.91 ± 0.78	5.9 ± 0.75	0.45
Social competencies					
Overall social competencies	5.77 ± 0.61	5.7 ± 0.55	5.73 ± 0.65	5.86 ± 0.61	0.59
General social competence	6.09 ± 0.67	6.12 ± 0.52	6.06 ± 0.73	6.1 ± 0.75	0.96
Performance in front of a group	5.86 ± 0.76	5.81 ± 0.69	5.86 ± 0.85	5.9 ± 0.75	0.89
Communication	5.94 ± 0.66	5.89 ± 0.52	5.83 ± 0.71	6.1 ± 0.7	0.17
Handling comments and questions	5.64 ± 0.86	5.5 ± 0.95	5.6 ± 0.74	5.81 ± 0.91	0.41
Feedback	5.57 ± 0.88	5.5 ± 0.99	5.63 ± 0.81	5.55 ± 0.89	0.99
Communication between tutor and mentor	5.8 ± 0.98	5.62 ± 0.9	5.8 ± 1.08	5.97 ± 0.91	0.37
briefing	5.47 ± 0.92	5.5 ± 0.99	5.34 ± 0.87	5.58 ± 0.92	0.52
Motivation					
Overall motivation	5.83 ± 0.71	5.88 ± 0.68	5.83 ± 0.73	5.78 ± 0.75	0.87
Tutoring	6.12 ± 0.88	6.19 ± 0.8	6.09 ± 0.95	6.1 ± 0.87	0.94
New expertise	6.11 ± 1.03	6.15 ± 1.08	6.11 ± 0.93	6.07 ± 1.12	0.92
Deepening ultrasound diagnostics	6.34 ± 0.86	6.5 ± 0.51	6.2 ± 1.08	6.36 ± 0.8	0.86
Commitment in addition to studies	5.91 ± 1.16	6.23 ± 0.95	5.97 ± 1.07	5.58 ± 1.34	0.13
Teach fellow students	6.41 ± 0.74	6.46 ± 0.71	6.46 ± 0.78	6.32 ± 0.75	0.62
Making contacts	5.79 ± 1.3	5.58 ± 1.45	5.86 ± 1.12	5.9 ± 1.38	0.56
Career development	5.13 ± 1.51	5.08 ± 1.5	5.23 ± 1.57	5.07 ± 1.5	0.89
Networking	5.42 ± 1.34	5.35 ± 1.2	5.46 ± 1.48	5.45 ± 1.34	0.76
Improve competencies	6.62 ± 0.71	6.77 ± 0.51	6.51 ± 0.78	6.61 ± 0.76	0.39
Earn money alongside your studies	4.44 ± 1.93	4.5 ± 1.05	4.46 ± 1.92	4.36 ± 1.91	0.93

The average overall didactic (5.33 ± 0.82), specific ultrasound (5.74 ± 0.62), and social competencies (5.77 ± 0.61) were self-evaluated in a scale range between 5.3 and 5.9 SP and remained consistent over time. This also applies to the subitems surveyed, which were evaluated in the range between 4.7 and 6.2 SP.

On average, the didactic competencies were self-evaluated significantly (*p* < 0.01) lower than the specific ultrasound and social competencies; this also tended to be the case per semester. The subitems of the didactic competencies “topic presentation,” “lesson introduction” and “lesson design” were rated significantly (*p* < 0.04) lower in Semester 13 than in the other semesters. In Semester 14, the competencies “carrying out tests” (*p* = 0.03) and “use of learning materials during course lessons” (*p* = 0.001) were evaluated significantly higher than in Semester 13. No significant differences per item could be measured over time within the subitems of specific ultrasound and social skills.

The results of the tutor’s self-reported motivations of various aspects are also presented in Table 3. The results ranged consistently between 4.4 and 6.8 SP without significant differences per item over the semesters under review. Particularly high evaluation results were recorded in the areas “improve competencies” and “teach fellow students,” while the lowest values were measured in the items “career development” and “earn money alongside your studies.”

### Results of assessments 3 and 4: the theoretical knowledge and practical competencies of peer teachers

**Figure 5:**
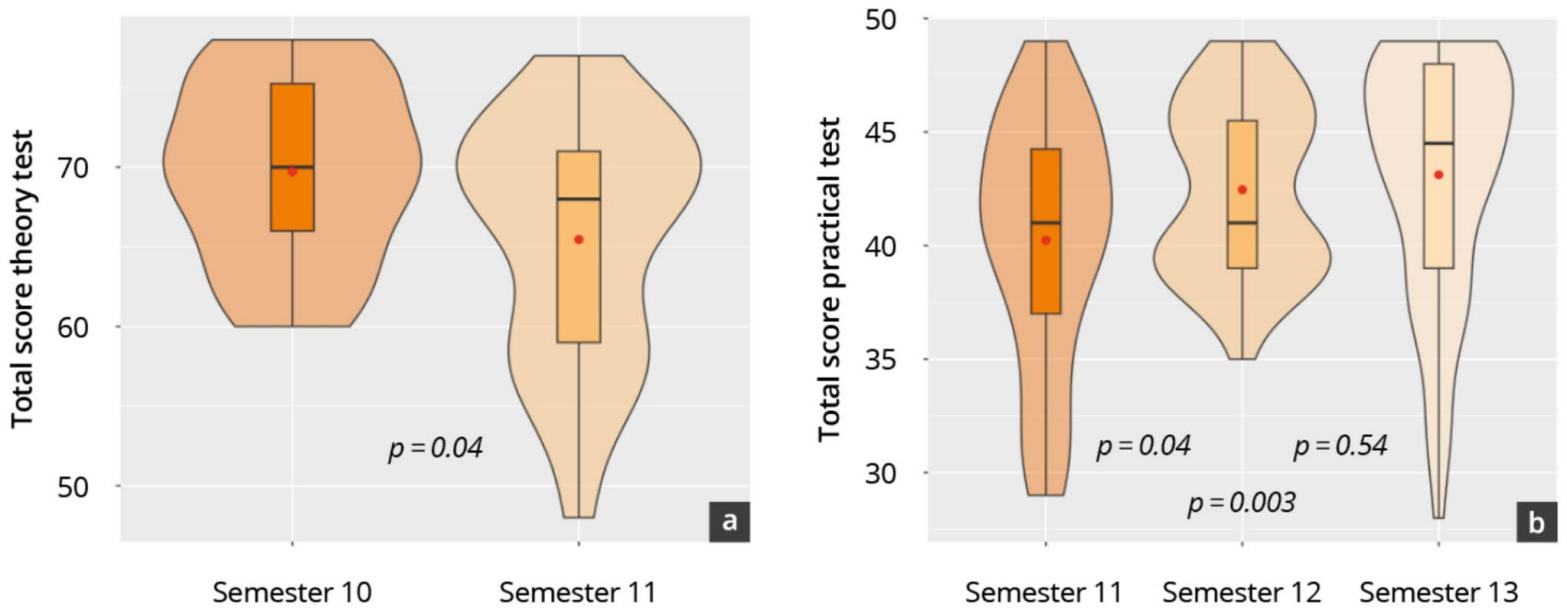
Results of the objective theory test **(A)** and practical test **(B)** in total for the various semesters.

A total of *N* = 44 theory test questionnaires and *N* = 147 practice test questionnaires were included in the evaluation from 2021 to 2024 (see Supplementary Tables 8, 9). Both the overall results of the theory test (67.2 ± 7.1 points) and the practice test (41.9 ± 5.1 points) remained consistently at a high level over the period under consideration, with some significant differences between the respective semesters. On the theory tests, significantly better scores were achieved in semester 10 than in semester 11 (*p* = 0.04, Figure 5), attributable to superior performance in the competence “normal findings.” A continuous increase in practical examination performance was recorded from semester 10 to semester 11.

### Comparison of results and correlations

**Figure 6:**
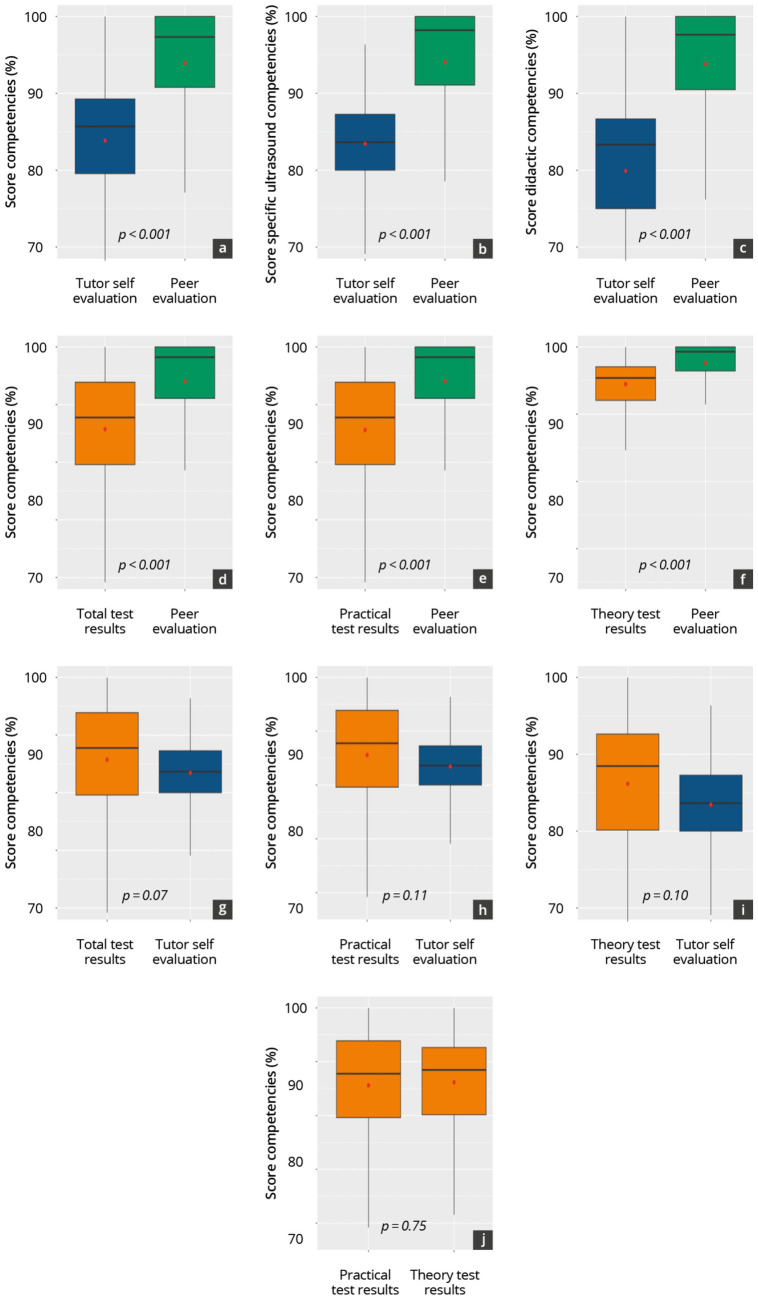
Comparison of the assessment results overall across all semesters. Comparison of the tutor self-evaluation with the peer evaluation **(a–c)**, the theory and practice test results with the peer evaluation **(d–f)**, the theory and practice test results with the tutor self-evaluation **(g–i)** and the theory and practice test results **(j)**.

Supplementary Table 10 and Figure 6 compare assessment results per semester and across semesters 1–14. The peer self-evaluations (assessment 1) of their specific ultrasound and didactic skills were significantly (*p* < 0.001) higher than the results of the peer-tutors’ self-evaluation (assessment 2) and the results of the theory and practical tests (assessments 3 and 4, respectively). Tutors tended to rate their specific ultrasound skills (assessment 2) lower than the results of the practical (assessment 4) and theoretical (assessment 3) tests, although the differences were not statistically significant. The theory test (assessment 3) results and practical test (assessment 4) results were consistently high, with no significant statistical differences between them.

## Discussion

This study evaluates the effectiveness of a peer-tutoring program designed to develop both theoretical and practical competencies in ultrasound instruction. The training concept was positively assessed across several measures, indicating that the quality of education of both peer tutors and their groups of learners was both achieved and maintained. Currently, there is a scarcity of dedicated peer-tutor curricula in the literature, underscoring the need for this program. Robust peer-tutoring training could ensure quality peer-to-peer teaching and ultrasound education more generally at universities.

Participants rated the quality of tutors highly, aligning with results from previous studies (46). Our questionnaire provided detailed assessments of both the professional and didactic qualities of the tutors. This positive feedback from peers indicates that the peer-tutoring approach is effective in developing competent educators. Stigmar et al. and Ten Cate et al. similarly found that peer-tutoring training significantly enhanced the teaching capabilities and content delivery skills of peer tutors (47, 48).

Tutors accepted the training concept and rated their competencies moderately. Overall self-ratings for key items remained consistent over time, except for the handling of teaching materials, which showed significant improvement across the training. This improvement may indicate continuous enhancement of the training concept. Tutors self-assessing positively is a good sign that they feel capable of delivering good instruction, as it reflects their confidence in both their subject matter expertise and their teaching abilities. This self-assessment reflects their belief in both their subject matter expertise and their teaching abilities. Confidence in these areas is crucial, as it often leads to more effective teaching. Tutors who feel capable are more likely to engage students successfully, adapt to different learning needs, and present material in a clear and understandable way.

The increased ratings for handling teaching materials suggest that tutors became more adept at using educational resources effectively, an area highlighted by Fellmer et al. and Menezes et al. as essential for successful instructional outcomes (49, 50).

Initially, the handling of teaching materials was rated lower, possibly due to a lack of experience in their didactic application. Early exposure to complex sonographic topics and practical training without prior confidence in teaching may also have contributed to lower initial scores. This indicate a need for more integrated refresher courses and dedicated didactic training. Incorporating additional sessions focused on the effective use of educational tools and materials could further bolster tutor preparedness and efficacy. The lower initial ratings underscore the importance of continuous professional development and support for peer tutors, as emphasized in the literature (46, 51).

Theoretical competencies showed significant improvement, with average scores in the competency-based categories remaining high (6.62 ± 0.71). These findings are consistent with prior research demonstrating the effectiveness of peer tutoring in enhancing theoretical knowledge (28, 42, 52). Studies by Alexander et al. and Shenoy et al. support the notion that peer-tutoring programs effectively enhance understanding and retention of complex theoretical concepts. The sustained high scores across semesters demonstrate the reliability of our peer-tutoring model in facilitating deep learning and comprehension (18, 53).

Practical competencies, assessed through tools like the total score of DOPS, also showed significant improvement, with scores averaging 41.94 ± 5.14 in the most recent semester (*p* = 0.009). This finding is in line with studies by Brierley et al. and Furmedge et al. (54, 55), which found that practical skills are even significantly enhanced through peer-led trainings for peer-tutors. The improvement in practical skills reflects the hands-on nature of the training, which is essential for developing competence in ultrasound techniques. The alignment between subjective evaluations and objective skill assessments suggests that participants perceived their learning progress accurately, an important marker of effective self-regulated learning (56). The practical training component, including the use of simulators and real-world applications of ultrasound, ensured that students were well-prepared for clinical scenarios, echoing the findings of Naeem et al. (57).

The results of the peer evaluation (assessment 1) were generally more favorable than the tutor self-evaluation (assessment 2) and the theoretical and practical results (assessments 3 and 4). This discrepancy might be due to the timing of evaluations—peer assessments occurred at the end of the course, while tutor self-assessments and practical evaluations were conducted at the beginning. This could indicate that tutors develop further during the course, as suggested by developmental progression theories in instructional training (58, 59). The timing of evaluations plays a crucial role in understanding the progression of competencies, and our findings highlight the dynamic nature of skill development over time. The discrepancy could also be explained by possible influencing factors such as previous teaching experience, self-confidence and social desirability. Tutors may perceive themselves more critically due to their awareness of areas for improvement, whereas students tend to evaluate on the basis of their overall learning experience. Future studies could provide further insights into these differences in perception through qualitative interviews or focus groups.

The congruence between theory and practical results and tutor self-assessment of competencies demonstrates that tutors’ self-evaluation corresponds with their objective skills, further suggesting the likelihood the training program was effective. The ability of tutors to accurately assess their skills is indicative of their reflective practice, which is essential for continuous improvement and professional growth. The self-awareness of tutors regarding their strengths and areas for development aligns with the findings of Celebi et al., who emphasized the importance of accurate reflective practice in effective education (32, 36).

Tutors’ self-reported motivations may play a crucial role in their effectiveness and commitment. In this study, particularly high scores were observed for the motivations of improving skills and teaching fellow students, while the lowest scores were recorded for career development and earning money while studying. These findings are consistent with previous research suggesting that intrinsic motivation is a key determinant of success in tutoring programs. Engels et al. identified several intrinsic motivational factors among peer tutors, including helping others, self-improvement, feedback and financial aspects (60). Our findings support the notion that peer tutors not only value opportunities for personal skill development, but also find fulfillment in contributing to the learning experiences of others. Although financial incentives ranked lower in importance, they still play a role in maintaining long-term commitment to tutoring programs. These findings should be considered in future program optimizations to improve tutor engagement and overall performance.

Published studies on tutor training have varied in duration and content, often lacking components like simulator training or e-learning (32, 36, 42). As Nguyen et al. found, however, the inclusion of technology-enhanced learning tools, such as apps and simulators, can provide interactive and engaging learning experiences (61), and thus training peer tutors in the use of mixed teaching media is important. Our program’s inclusion of diverse instructional tools and comprehensive training modules, including simulator and app-based learning, provides a more holistic approach than those of prior studies. The comprehensive nature of our program ensured that tutors were well-equipped with both theoretical knowledge and practical skills. Future programs should emphasize these areas, particularly the integration of advanced didactic training.

There is potential for future improvements to the training program developed here, including stronger national and international standardization of peer-tutoring curricula.

The structured peer-tutor training curriculum outlined in this study could be adapted for other medical specialties by integrating discipline-specific theoretical and practical modules while maintaining core pedagogical principles (46). For instance, specialties such as cardiology, radiology, or emergency medicine could incorporate targeted ultrasound training, while surgical fields could focus on procedural simulations (62, 63). Standardized frameworks for peer-tutor training should emphasize competency-based education, incorporating objective assessments such as direct observation of procedural skills (DOPS) and validated theoretical examinations. To facilitate international standardization, professional societies and accreditation bodies should collaborate to establish consensus guidelines defining peer-tutor qualifications, minimum training durations, and certification criteria. Creating an internationally recognized certification pathway could enhance peer tutoring’s credibility and promote the integration of structured peer education across diverse medical curricula (64). Future research should explore the long-term impact of standardized peer-tutor training on clinical competency and knowledge retention across various medical disciplines.

While it is true that a positive self-assessment by tutors can indicate confidence and potential teaching efficacy, a more robust evaluation would involve comparing the outcomes of trained peer tutors against those of untrained peer tutors or expert academic tutors. This comparative approach would provide a more objective assessment of the effectiveness of tutor training programs. Standardization can ensure consistency in training quality and outcomes, facilitating the comparison and replication of successful models across institutions. The establishment of certification for tutors, recognized by professional societies, could further enhance the credibility and attractiveness of peer-tutoring programs. Such certification can serve as a mark of excellence, motivating tutors to engage in continuous professional development and justifies the deployment of non-experts in university teaching in marketized higher education contexts. As suggested by Höhne et al., certification and standardization can also provide a framework for evaluating and benchmarking tutor competencies, contributing to the overall improvement of educational programs (43).

### Limitations

While this study provides valuable insights into the effectiveness of the ultrasound peer-tutor training curriculum, several limitations should be acknowledged. Firstly, the participant evaluations of tutor competencies were very positive, but there was no comparison with instruction provided by medical professionals, such as physicians (65, 66). This absence of a control group means we cannot definitively attribute the high evaluations solely to the peer tutor training program. Secondly, the study did not employ objective measurement instruments to assess the didactic skills of the tutors. Future studies should incorporate such objective tools to provide a more comprehensive evaluation of the tutors’ teaching abilities. Thirdly, the study did not include subgroup analyses or individual assessments of the tutors due to the small sample size. This limitation restricts our understanding of the variability in performance among individual tutors and the specific factors that might contribute to their effectiveness.

Additionally, data collection occurred across different semester time points (at the beginning and end) across semesters, potentially introducing variability in the results due to changes in curriculum implementation or student cohorts. This temporal variability could impact the consistency and comparability of the findings. Finally, the small sample size also precluded a detailed analysis of individual participants’ progress and outcomes. A larger sample size in future studies would enable a more granular examination of the effectiveness of the training program for individuals.

## Conclusion

This study developed, implemented, and evaluated an ultrasound peer tutor-training curriculum. Results suggest that it was effective in preparing peer tutors for their teaching roles. The structured, comprehensive approach, encompassing 11 modules over a year, ensured high-quality training in theoretical knowledge and practical skills as measured in objective tests and subjective self-and peer reports, and gave peer tutors confidence in deploying didactic techniques. Grounded in interdisciplinary collaboration and current literature, the design of the curriculum addresses the current challenges of ultrasound education, particularly university hospitals’ common constraints on available resources, time, and staff.

The consistently high evaluations from peer-tutored course participants and the stable self-assessments from peer tutors highlight the curriculum’s popularity. Despite the positive outcomes, the study is limited by a lack of a control group for comparison with physician-led instruction, the absence of objective measures of peer tutors’ didactic skills, and the inability to perform subgroup analyses due to the study’s small sample size. These limitations suggest areas for future research, where studies could incorporate objective assessment tools for peer-tutor teaching, test the concept among a larger peer-tutor sample size, and compare the peer-tutor model with traditional physician-led teaching methods.

Overall, the study underscores the attainability and effectiveness of peer-tutor training to ensure quality ultrasound education, and it highlights the need for standardization in peer-tutor training programs. The findings indicate the need for further research and collaboration to establish national and international guidelines for peer tutoring, ensuring consistency and quality across institutions and global contexts. By addressing the challenges faced by its teaching institutions globally, medical education can continue to advance, providing high-quality, practical, and theoretical training to future healthcare professionals.

## Data Availability

The raw data supporting the conclusions of this article will be made available by the authors, without undue reservation.

## Footnotes

^1^ http://www.rstudio.com

^2^ http://www.R-project.org
