# Supplementary material for: Quality assessment of a training program for undergraduate sonography peer tutors: paving the future way for peer-assisted learning in medical ultrasound education
Source: Front Med (Lausanne). 2025 Mar 3;12:1492596. doi: 10.3389/fmed.2025.1492596 (PMC11911324; doi:10.3389/fmed.2025.1492596)
Supplement: Supplementary file 1 [file Data_Sheet_1.pdf]

Supplement 1: Structure and content of the didactics lecture notes for tutor training

Aspect	Content	Educational goals
Didactic tips	• Participant questions<br>• Didactic tools	• Handling participant questions<br>• Feedback tips/feedback rules<br>• Teaching tips<br>• Presentation tips<br>• Avoiding potential problems/mistakes<br>• Characteristics of course quality
Lesson preparation	• Different teaching media	• Tips for lesson preparation/review<br>• Promoting motivation<br>• Blackboard layout tips<br>• Tips for using different teaching media
Station training	• Different practices<br>• Device settings	• Ultrasound image formation<br>• Deriving standard planes<br>• Intuitive transducer handling<br>• Better verbalization<br>• Improved transducer guidance<br>• Use of the "Scan Coach"<br>• Creation and presentation of Case studies
