# Supplementary material for: Quality assessment of a training program for undergraduate sonography peer tutors: paving the future way for peer-assisted learning in medical ultrasound education
Source: Front Med (Lausanne). 2025 Mar 3;12:1492596. doi: 10.3389/fmed.2025.1492596 (PMC11911324; doi:10.3389/fmed.2025.1492596)
Supplement: Supplementary file 2 [file Data_Sheet_2.pdf]

Supplement 2: Feedback form for tutors

To maintain and improve the quality of teaching, we would like to provide feedback to the tutors through this questionnaire.
We therefore ask you to evaluate the teaching in selected areas. Please rate the performance shown using a grading scale (A / A- / B+ / B / B- / etc.) and circle the elements to which your observations mainly refer. Additionally, we would appreciate it if you could write down your ideas for improvement as a tip.

1 Introduction: e.g. learning goals presented briefly and concisely, motivation for the lesson, lesson introduction

Tips:____

2 Explanation of standard planes and examination procedure: e.g. use of posters/PowerPoint/blackboard layout

Tips:____

3 Final summary: e.g. reviewed learning objectives, motivation for learning, preview of the next lesson

Tips:____

4 Interaction with the group:

Tips:____

5 Appreciative acknowledgment: e.g. eye contact, varied signals, reasoning, fairly distributed

Tips:____

6 Handling questions: e.g. repeated, considered/deferred in the course, answered concisely, involved the group, reassured

Tips:____

7 Precise wording: anatomical spatial relationships, clear formulation of questions, technical terms (cranial, caudal/"above, below"), ultrasound guidance

Tips:____

8 Eye contact: e.g. attention to everyone, alternating between participants, shifting between participants/monitor/board

Tips:____

9 Speaking time:

Tips:____

10 Authority:

Tips:____

11 Position in the room: e.g. the position itself, ensures clear sight lines, notices issues with participants

Tips:____

12 Guidance with the ultrasound probe: e.g. precise instructions, demonstrates correct probe positioning, shows correct pressure, intervenes when necessary

Tips:____

13 Including "non-scanning" course participants: e.g. targeted task distribution, use of teaching materials, resolving questions

Tips:____

14 Authentic questions: e.g. open-ended questions, questions without a "testing" tone

Tips:____

15 Handling ultrasound equipment: e.g. using the trackball, explaining anatomy/allowing explanation, addressing ways to improve the image, using pointers, labeling the image

Tips:____

16 Use of teaching materials (whiteboard, flashcards, educational software, flip chart, PowerPoint, live transmission)

Tips:____

17 Other observations and general tips:

____
