# Supplementary material for: Quality assessment of a training program for undergraduate sonography peer tutors: paving the future way for peer-assisted learning in medical ultrasound education
Source: Front Med (Lausanne). 2025 Mar 3;12:1492596. doi: 10.3389/fmed.2025.1492596 (PMC11911324; doi:10.3389/fmed.2025.1492596)

Supplement 3 Sample questions of the theory-test (assessment 3)

Sample question ultrasound basics 1

- Which imaging modes are shown here?

a

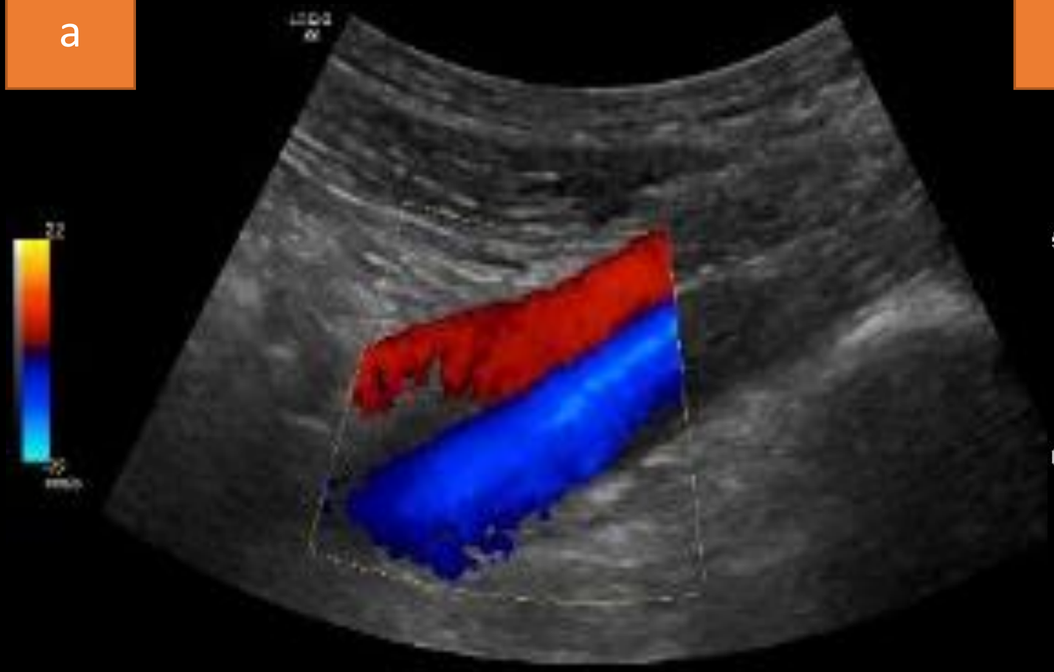

b

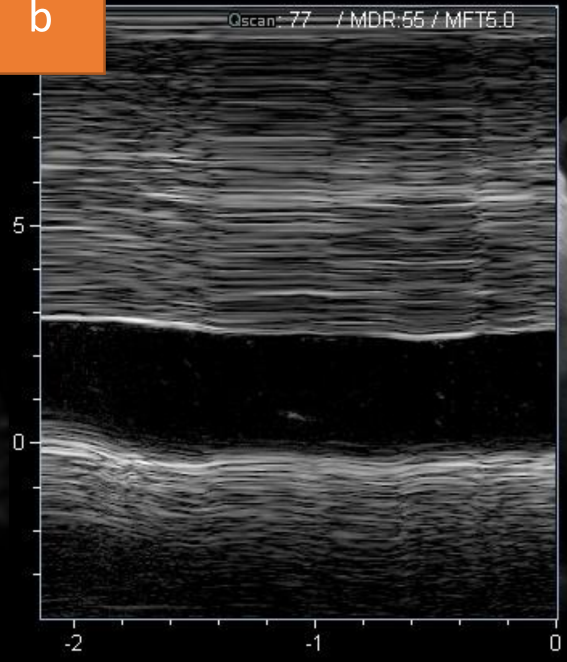

c

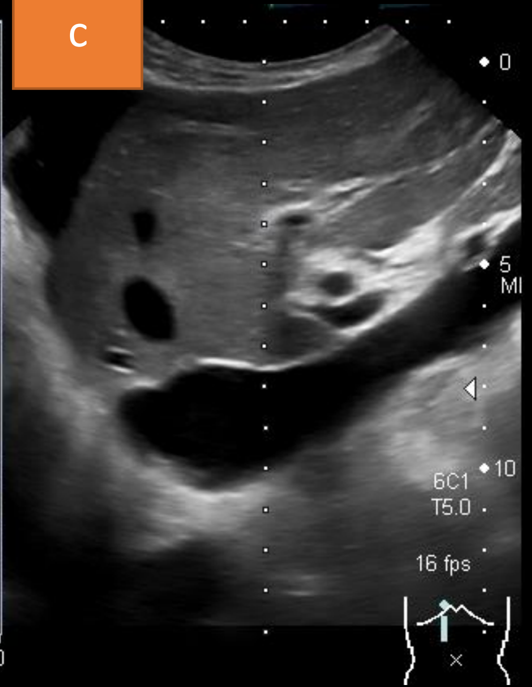

Sample question ultrasound basics 2

- Which artifacts are marked with arrows here?

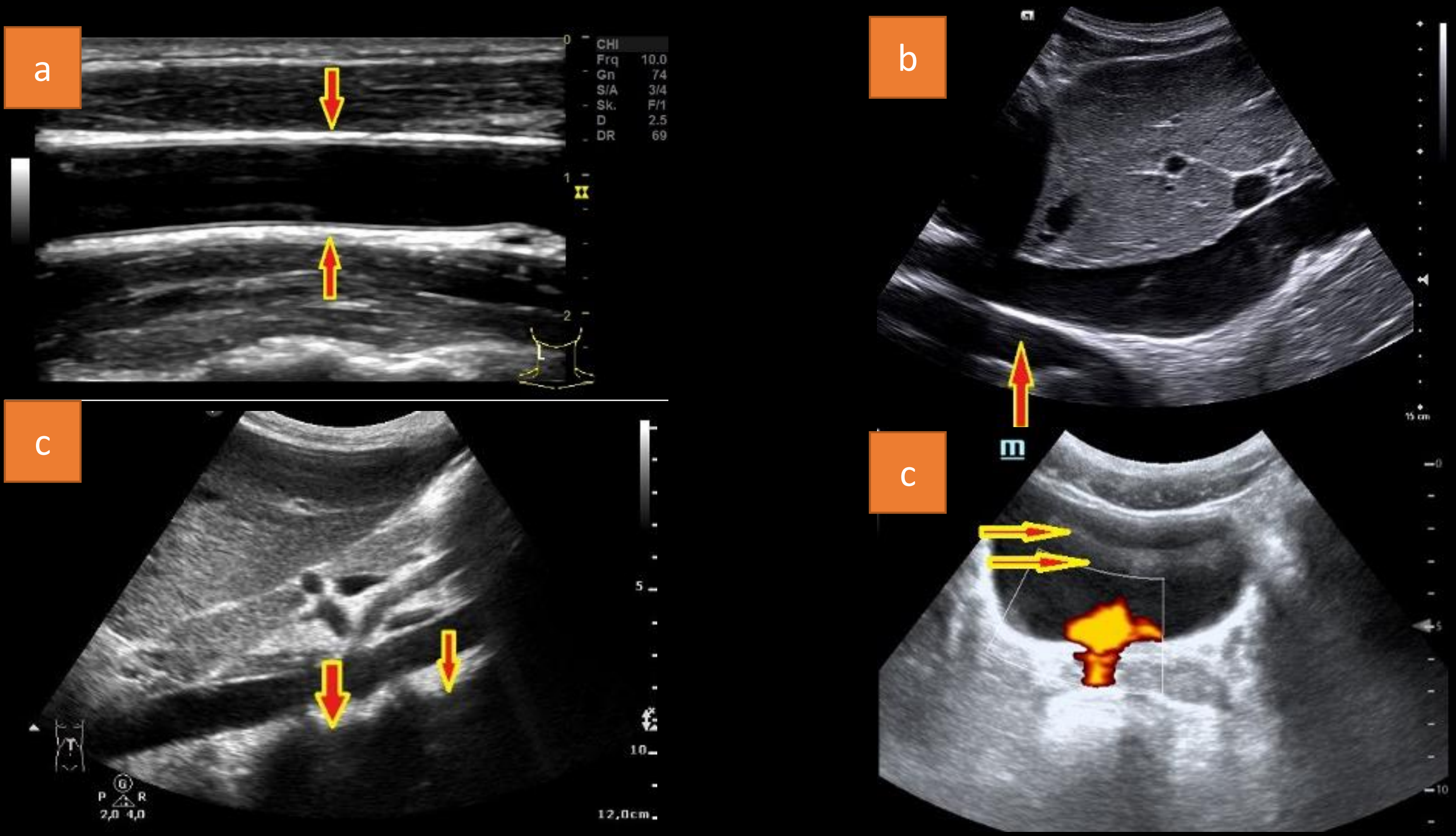

Sample question basics 3

- Label the image with the correct anatomical directions. Pay attention to the ultrasound probe image provided.

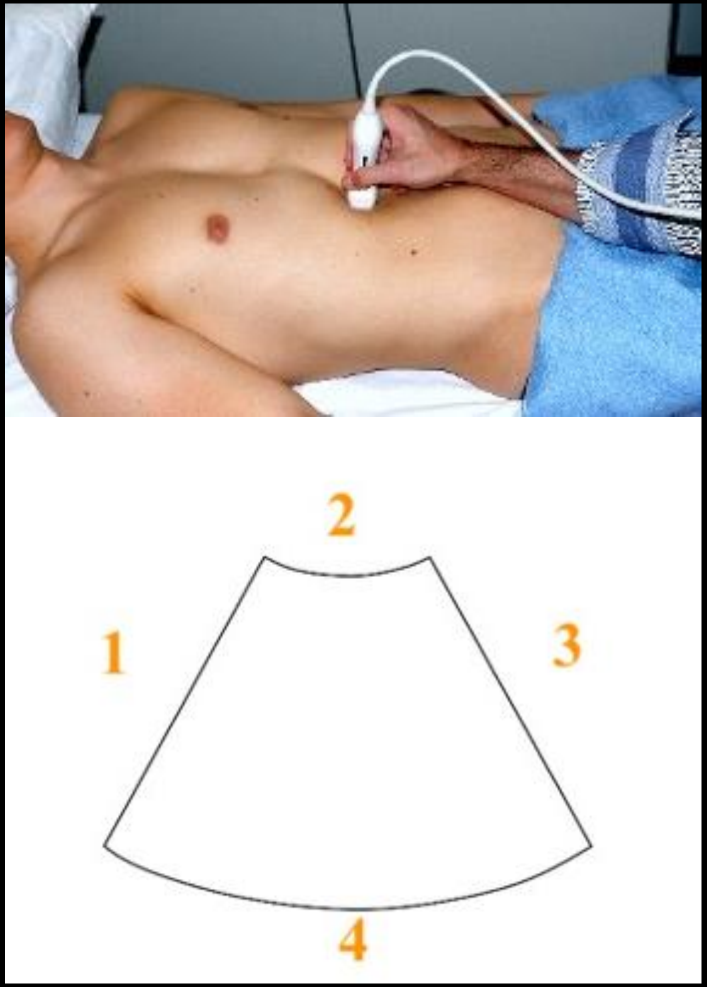

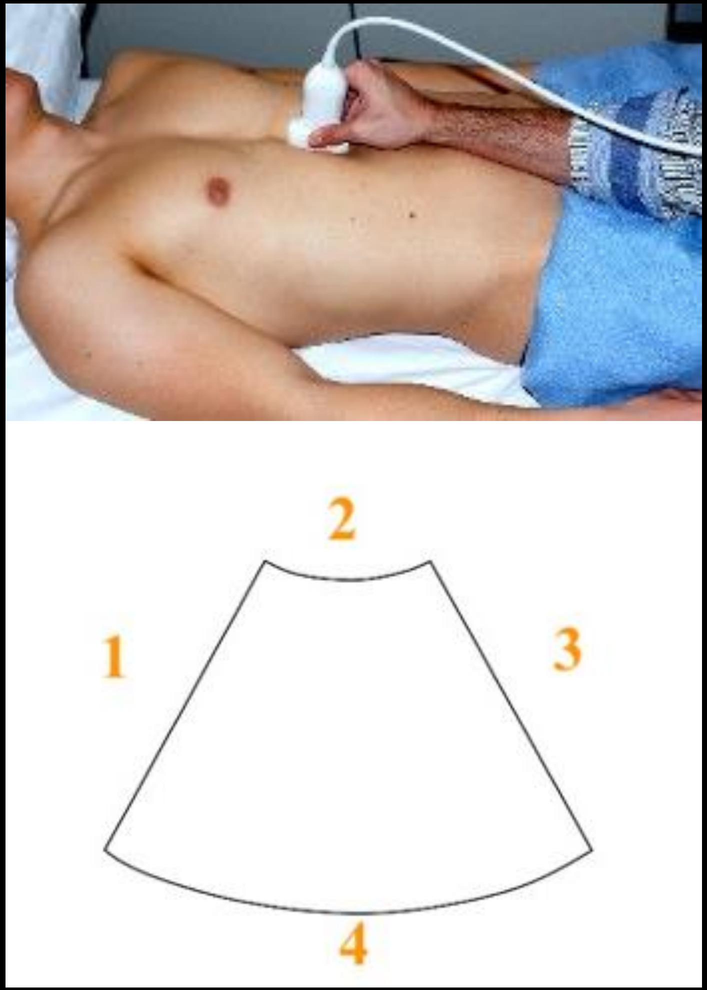

Sample question ultrasoundbasics 4

- Which of the following ultrasound images most closely matches the ultrasound probe image shown?

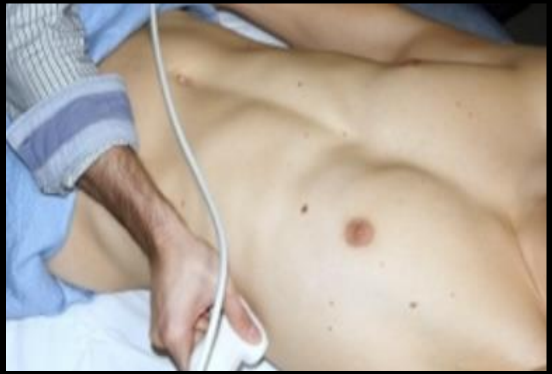

a

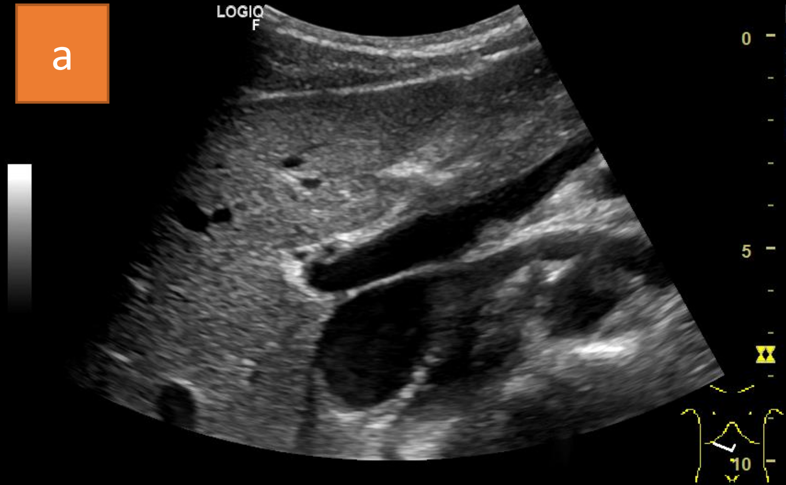

b

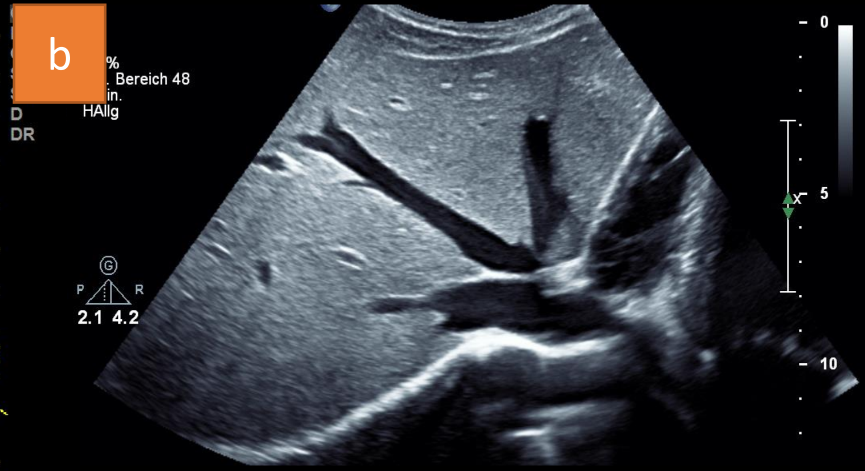

c

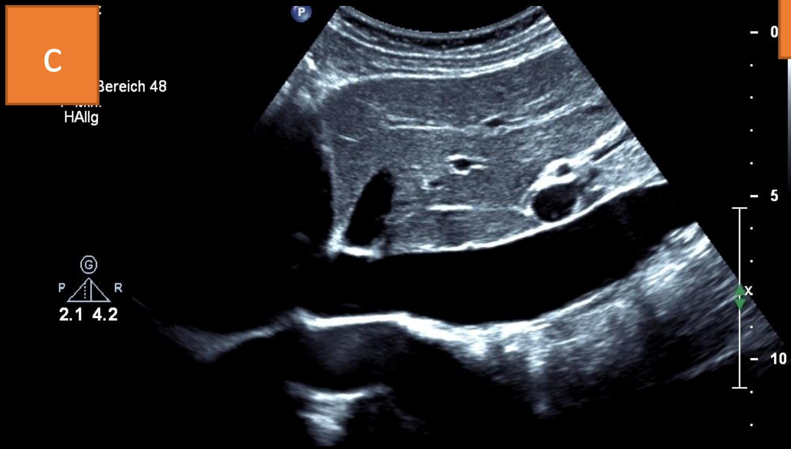

d

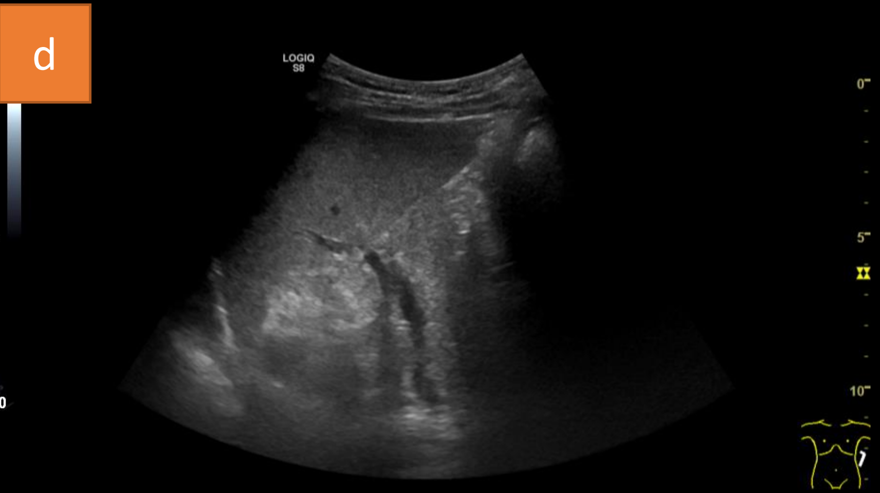

Sample question ultrasound basics 5

- Which of the following ultrasound probe images most closely matches the shown ultrasound image?

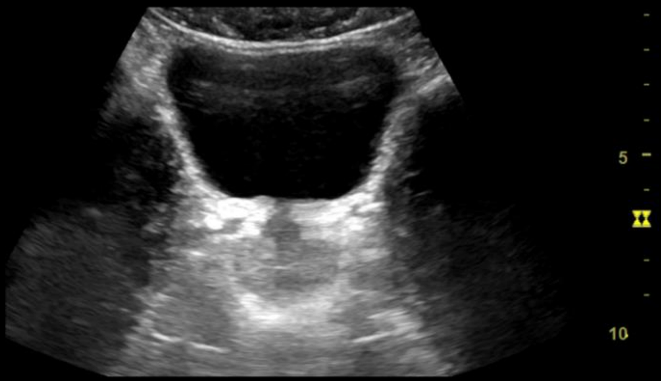

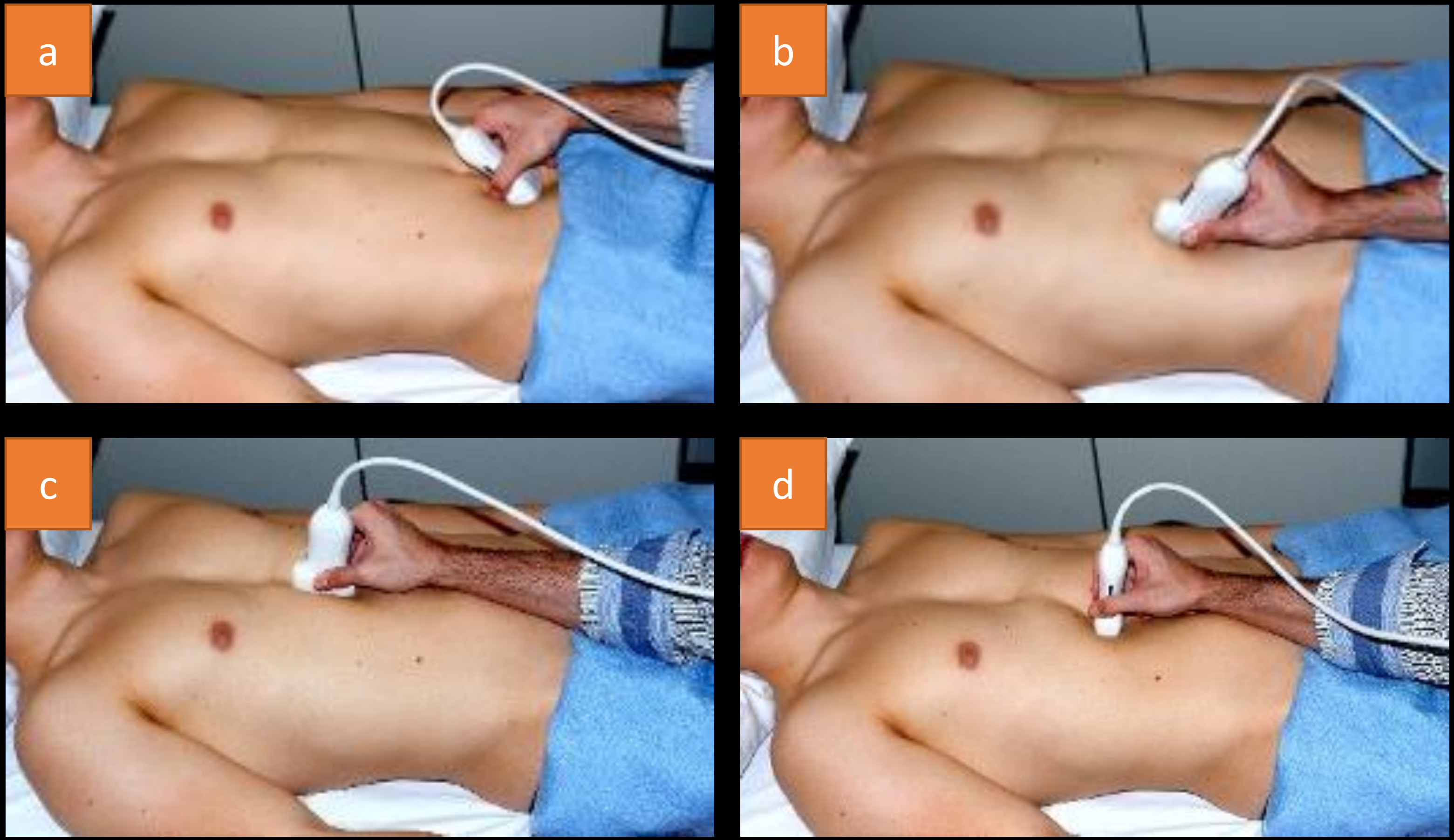

Sample question ultrasound normal findings 1

- Label the numbered structures as precisely as possible!

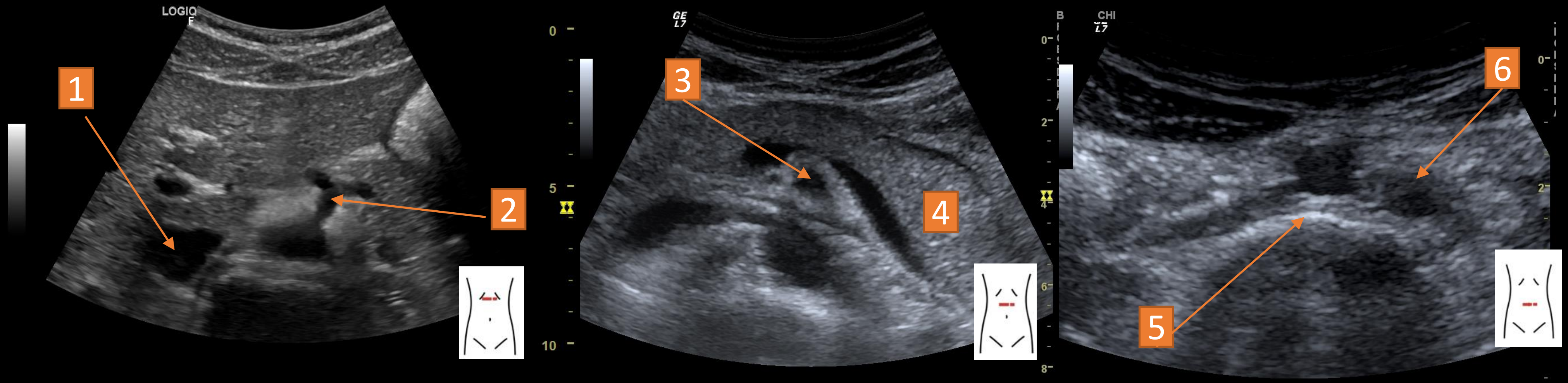

Sample question ultrsound normal findings 2

- Label the numbered structures as precisely as possible!

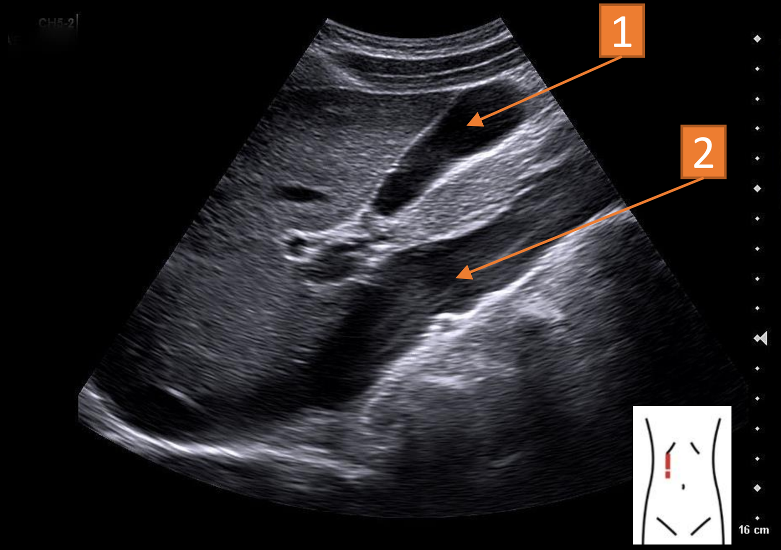

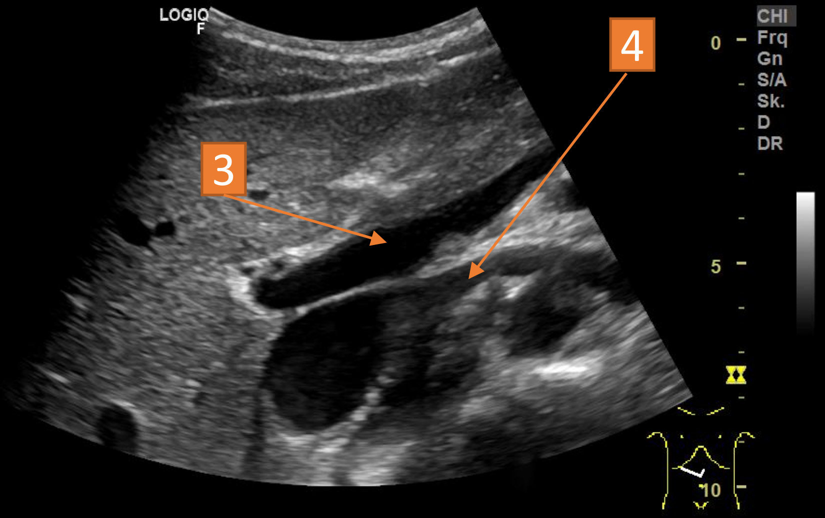

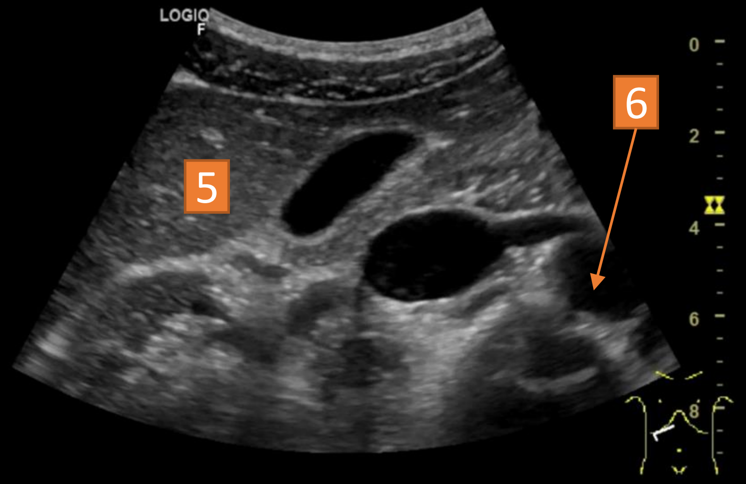

Sample question ultrasound normal findings 3

- Label the numbered structures as precisely as possible!

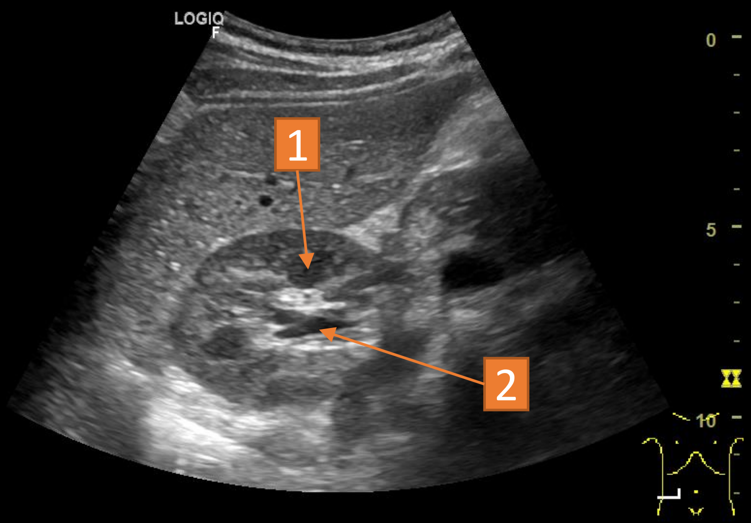

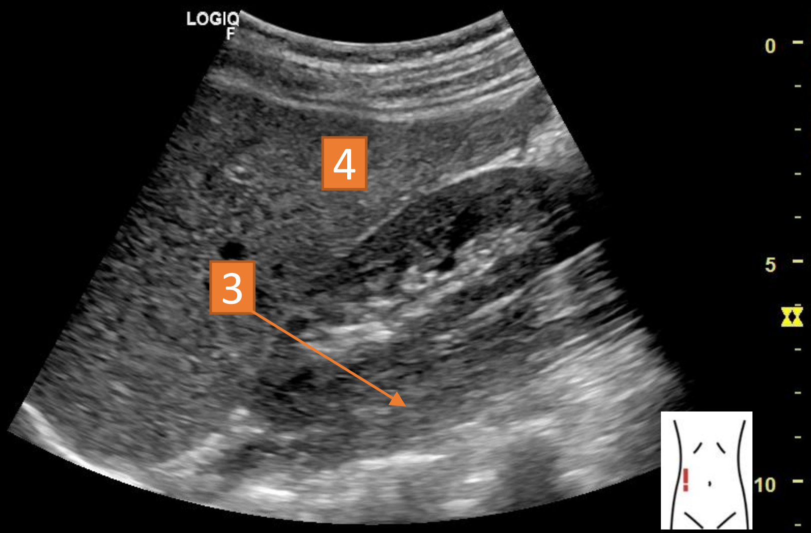

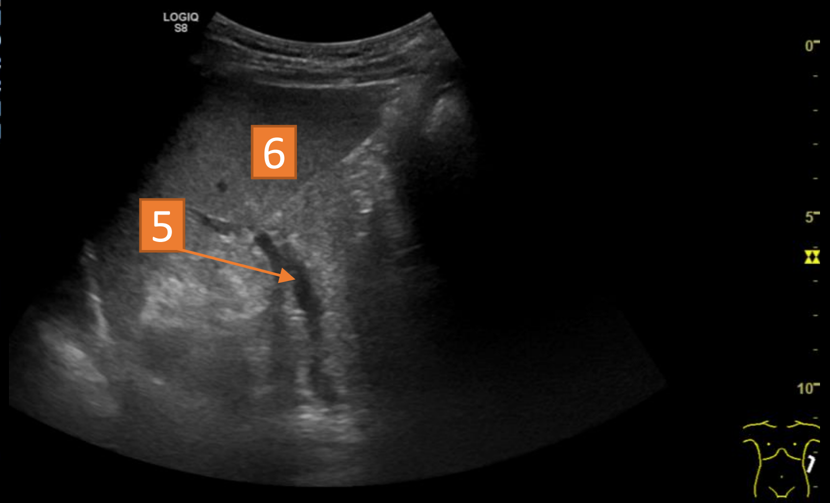

Sample question ultrasound normal findings 4

- Label the numbered structures as precisely as possible!

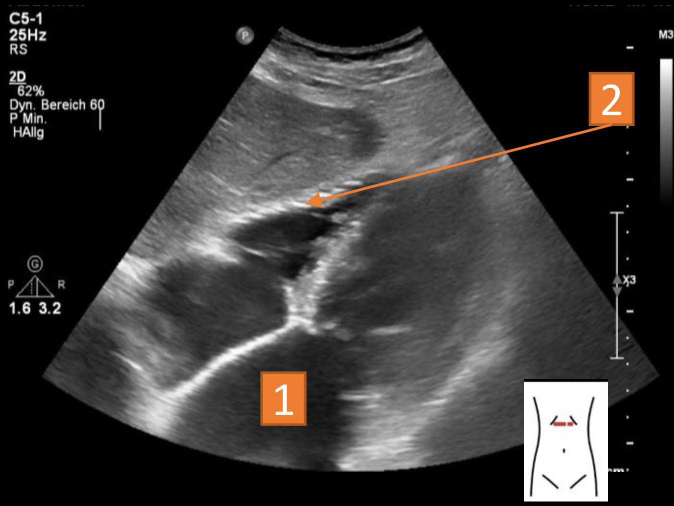

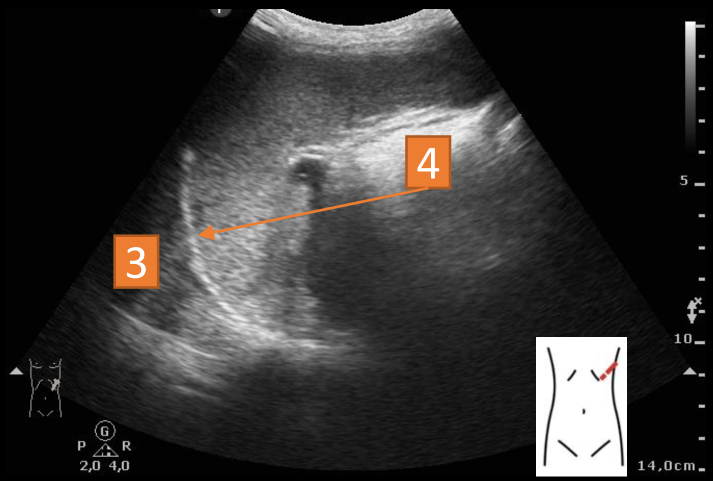

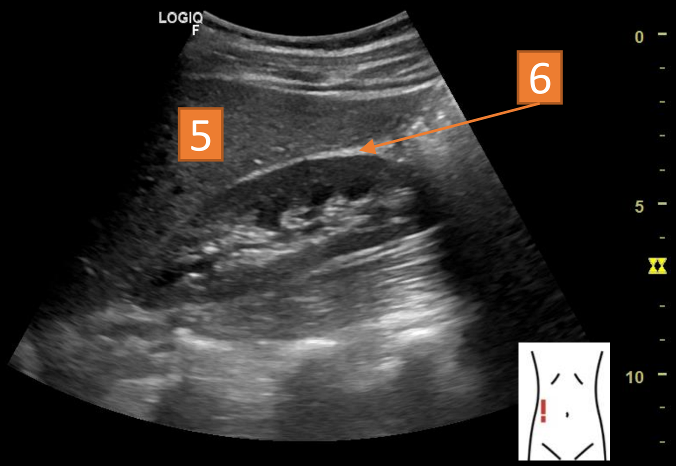

Sample question ultrasound pathological findings 1

- What do the depicted pathologies most likely represent?

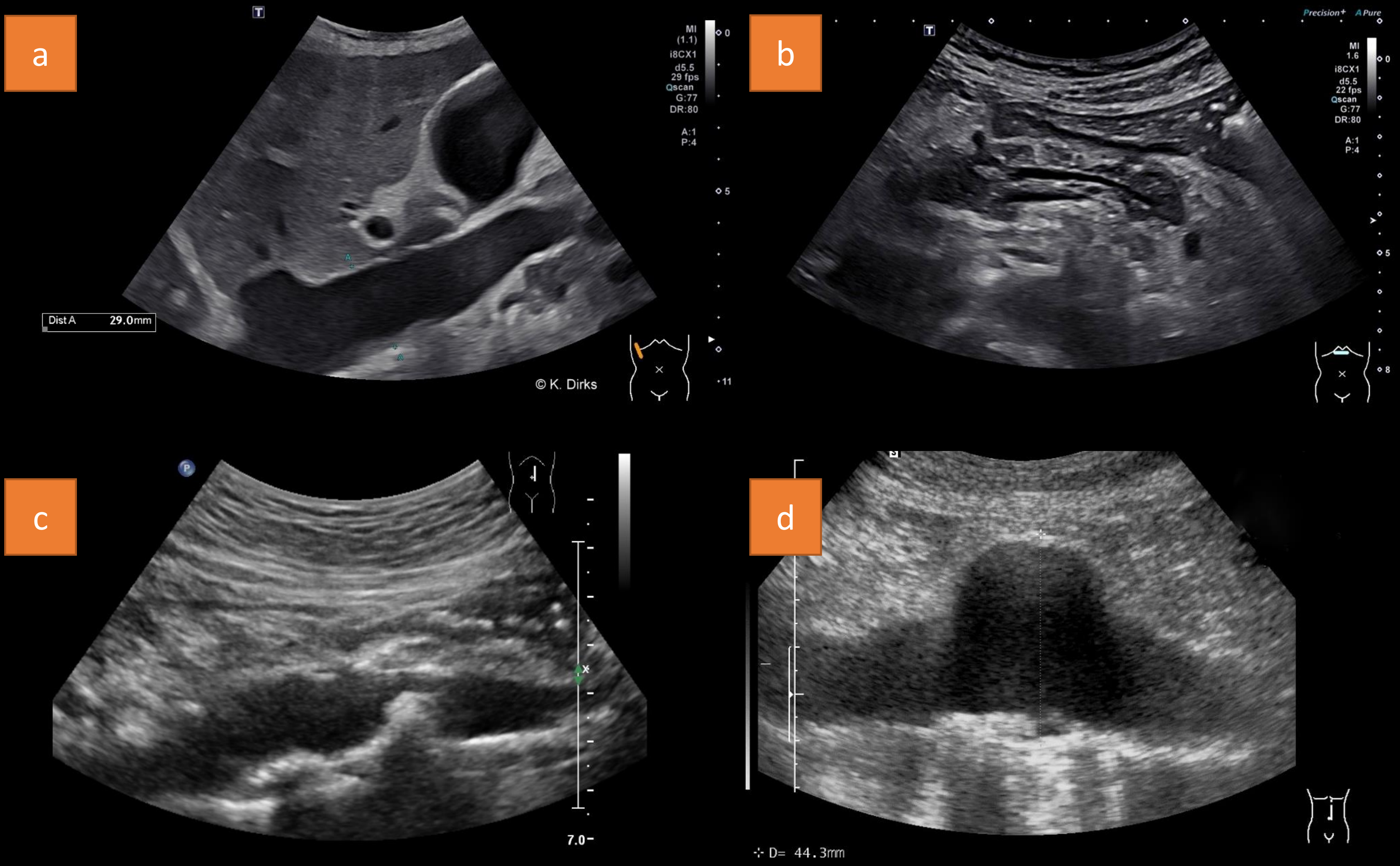

Sample question ultrasound pathological findings 2

- What do the depicted pathologies most likely represent?

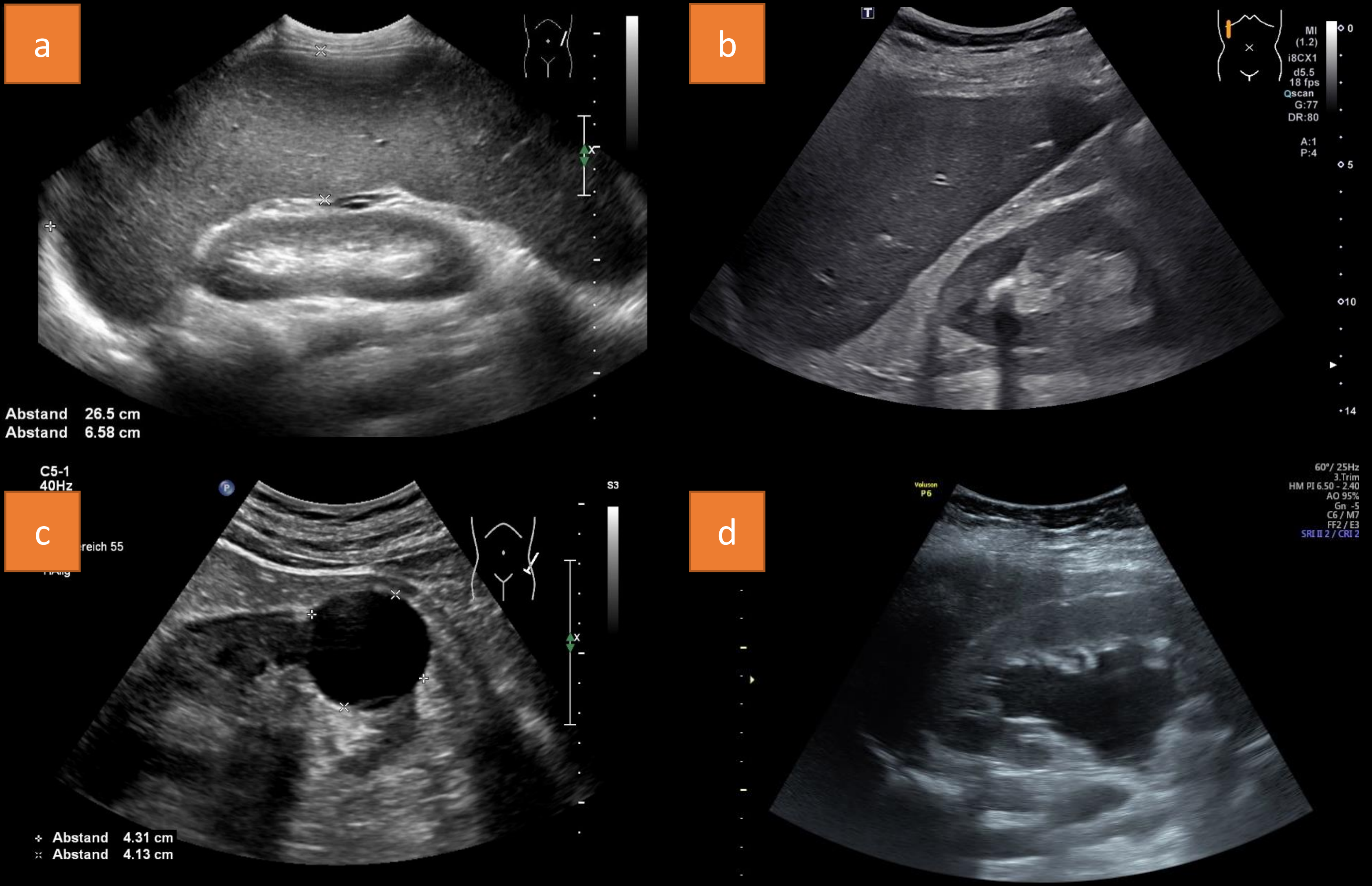

Sample question ultrasound pathological findings 3

- What do the depicted pathologies most likely represent?

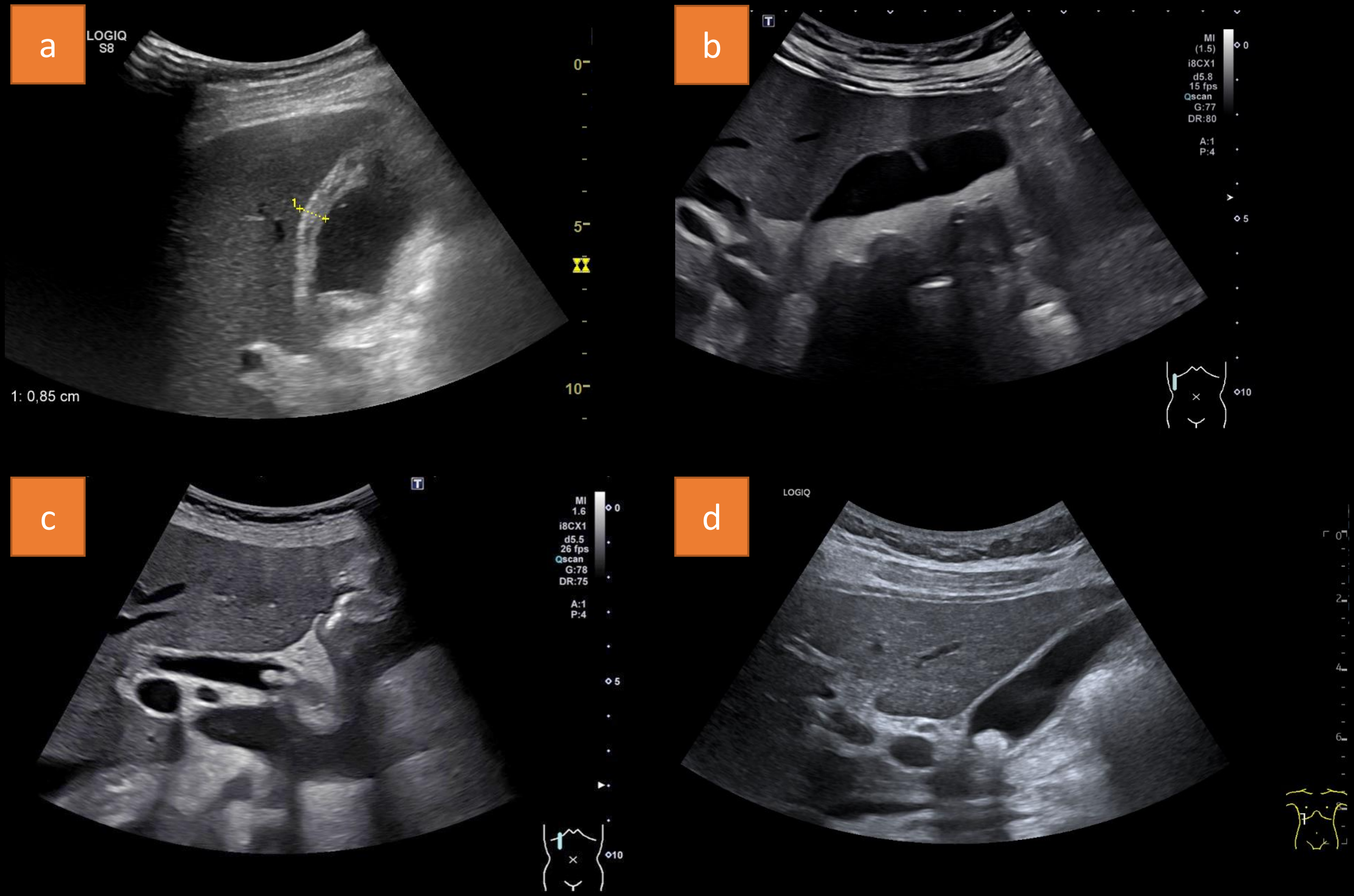

Supplement: Supplementary file 3 [file Data_Sheet_3.pdf]
