# Supplementary material for: Quality assessment of a training program for undergraduate sonography peer tutors: paving the future way for peer-assisted learning in medical ultrasound education
Source: Front Med (Lausanne). 2025 Mar 3;12:1492596. doi: 10.3389/fmed.2025.1492596 (PMC11911324; doi:10.3389/fmed.2025.1492596)
Supplement: Supplementary file 4 [file Data_Sheet_4.pdf]

**Supplement 4:** Example examination sheet

**DOPS No. ________: Topic _______ – evaluation form**

Code participant:_________ examiner:___________

**Task 1:** Guide the patient appropriately through the examination. Name possible **indications / questions**.

| **Patient management / Communication/ Indications** (max. 10 Credits) | | | |
|---|---|---|---|
| **Establishing a relationship**<br>Greeting, introducing yourself ,asking for name; asking about the patient's feeling | 2 | 1 | 0 |
| **Patient preparation**<br>Naming the occasion; asks about previous experiences; name the procedure and obtaining consent; indicates sufficient undressing; warning of sonic gel and light pressure | 2 | 1 | 0 |
| **Communication during the examination**<br>Empathic communication; pays attention to non-verbal and para-verbal communication; explains own actions and need for co-operation; gives clear instructions and understandable information | 2 | 1 | 0 |
| **Gives inhale & exhale command if required** | 2 | 1 | 0 |
| Names **examination indications and questions** | 2 | 1 | 0 |

**Task 2:**
Please **scan the entire** [...] and **show** the following **anatomical structures / landmarks**: [...]

| **Transducer-handling** (max. 8 Credits) | |
|---|---|
| **Orientation** | |
| Correct or immediately self-checked based on image movement / by uncoupling | 2 |
| Corrected after initial difficulties / after prompting | 1 |
| Correct orientation only found with manual help | 0 |
| **Positioning** | |
| Correct or immediately transferred from another section | 2 |
| Corrected after initial difficulties / after prompting | 1 |
| Correct orientation position only found with manual help | 0 |
| **Coupling / Transducer position** | |
| Transducer is coupled well with sufficient gel, continuous good pressure, good posture | 2 |
| Corrected after initial difficulties / after prompting | 1 |
| No pressure and/or no gel + discontinuous pressure and/or transducer uncoupled | 0 |
| **Adequate enlargement / image optimisation / device operation** | |
| Independent & adequate adjustment with appropriate image quality (gain, penetration depth, frequency, focus) even during examination, left hand on the device | 2 |
| Corrects after initial difficulties / after prompting, forgets optimisation during examination, does not keep left hand on the device | 1 |
| No adequate image setting despite request | 0 |

| **Screening** (max. 8 Credits) | | |
|---|---|---|
| · **Full sagittal examination** beyond structural limits, appropriate speed<br>· **Full transverse examination** beyond structural limits, appropriate speed | 4 | 4 |
| · Incomplete sagittal examination / inadequate speed / verbal help necessary<br>· Incomplete transverse examination / inadequate speed / verbal help necessary | 2 | 2 |
| · Sagittal examination not performed or performed only with manual assistance<br>· Transverse examination not performed or performed only with manual assistance | 0 | 0 |

| **Image explanation:** Correctly **pointing out and naming the structures** (max. 4 Credits) | | | | | | | | |
|---|---|---|---|---|---|---|---|---|
| Structure 1 | 1 □ | Structure 2 | 1 □ | Structure 3 | 1 □ | Structure 4 | 1 □ | (…) |

Example DOPS Abdominal Weimer et. al. 2024

**Task 3+4:** Please **measure** [...]/and **save** an **image/clip.**

| Measurement (max. 6 Credits) | | |
|---|---|---|
| · Correct measurement point in first sectional plane, orthogonal to organ/vessel wall<br>· Correct measurement point in second sectional plane, orthogonal to organ/vessel wall | 3 | 3 |
| · Inaccurate measurement point placement in first sectional plane / only performed with verbal help<br>· Inaccurate measurement point placement in second sectional plane / only performed with verbal help | 1 | 1 |
| · Measurement in first sectional plane only performed with manual help<br>· Measurement in second sectional plane only performed with manual help | 0 | 0 |
| **Image documentation:** correct saving oft he image (max. 1 Credit) | 1 | 0 |

**Task 5: Interpret** the submitted **examination report** after QR-code-scan and explain the **possible further procedure** (max. 4 Credits)

| | |
|---|---|
| Correct pathology identification without hints | 2 |
| No correct pathology identification | 0 |
| Correct naming/suggestion of further diagnostic/therapeutic procedure | 2 |
| Incorrect naming/suggestion of further diagnostic/therapeutic procedure | 0 |

**Overall impression**: I rate the overall impression with (max. 8 Credits – please circle)

1 – 2 – 3 – 4 – 5 – 6 – 7 – 8

**Total Score**: __/ 49
