# Supplementary material for: Quality assessment of a training program for undergraduate sonography peer tutors: paving the future way for peer-assisted learning in medical ultrasound education
Source: Front Med (Lausanne). 2025 Mar 3;12:1492596. doi: 10.3389/fmed.2025.1492596 (PMC11911324; doi:10.3389/fmed.2025.1492596)
Supplement: Supplementary file 5 [file Data_Sheet_5.pdf]

Supplement 5 Results of tutor competencies (assessment1) by course participants (peer group)

Item (Likert scale 1=very low, 7=very high))	Overall (N)	Semester 1	Semester 2	Semester 3	Semester 4	Semester 5	Semester 6	Semester 7	Semester 8	Semester 9	Semester 10	Semester 11	Semester 12	Semester 13	Semester 14
	N = 2987	N = 51	N = 151	N = 219	N = 229	N = 211	N = 290	N = 321	N = 115	N = 158	N = 154	N = 209	N = 306	N = 333	N = 240
**Overall competence**	6.6 ± 0.6	6.5 ± 0.4	6.2 ± 0.7	6.5 ± 0.5	6.6 ± 0.5	6.6 ± 0.4	6.6 ± 0.5	6.7 ± 0.4	6.7 ± 0.4	6.7 ± 0.4	6.7 ± 0.4	6.6 ± 0.5	6.7 ± 0.4	6.3 ± 1.0	6.5 ± 0.8
**Overall specific ultrasound competencies**	6.6 ± 0.6	6.4 ± 0.5	6.3 ± 0.7	6.5 ± 0.6	6.6 ± 0.5	6.6 ± 0.5	6.6 ± 0.5	6.7 ± 0.5	6.8 ± 0.3	6.8 ± 0.4	6.7 ± 0.4	6.6 ± 0.5	6.7 ± 0.4	6.3 ± 1.1	6.5 ± 0.9
Knowledge	6.4 ± 0.7	6.1 ± 0.8	5.9 ± 0.9	6.2 ± 0.8	6.4 ± 0.7	6.4 ± 0.7	6.4 ± 0.7	6.6 ± 0.6	6.7 ± 0.5	6.6 ± 0.6	6.5 ± 0.7	6.3 ± 0.8	6.6 ± 0.6	-	-
Use of the device	6.5 ± 0.6	6.1 ± 1.0	5.9 ± 1.1	6.3 ± 0.8	6.4 ± 0.7	6.5 ± 0.6	6.5 ± 0.7	6.7 ± 0.6	6.7 ± 0.6	6.6 ± 0.6	6.5 ± 0.7	6.4 ± 0.8	6.6 ± 0.6	-	-
Transducer handling	6.7 ± 0.6	6.6 ± 0.6	6.5 ± 0.7	6.6 ± 0.6	6.7 ± 0.5	6.8 ± 0.5	6.7 ± 0.6	6.8 ± 0.5	6.9 ± 0.3	6.8 ± 0.4	6.8 ± 0.5	6.6 ± 0.7	6.7 ± 0.5	-	-
Spatial orientation	6.7 ± 0.6	6.6 ± 0.6	6.3 ± 0.9	6.6 ± 0.7	6.6 ± 0.6	6.7 ± 0.5	6.7 ± 0.6	6.7 ± 0.6	6.9 ± 0.3	6.8 ± 0.4	6.7 ± 0.5	6.7 ± 0.6	6.7 ± 0.6	-	-
Sono-anatomical correlation	6.7 ± 0.6	6.6 ± 0.6	6.3 ± 0.8	6.5 ± 0.7	6.6 ± 0.6	6.7 ± 0.5	6.7 ± 0.5	6.8 ± 0.5	6.9 ± 0.4	6.8 ± 0.5	6.8 ± 0.5	6.6 ± 0.6	6.7 ± 0.5	-	-
Visualization of organs	6.7 ± 0.6	6.6 ± 0.5	6.4 ± 0.8	6.6 ± 0.6	6.7 ± 0.5	6.7 ± 0.5	6.7 ± 0.6	6.8 ± 0.5	6.9 ± 0.4	6.8 ± 0.5	6.8 ± 0.5	6.6 ± 0.7	6.7 ± 0.5	-	-
Examination and assessment of the organs	6.6 ± 0.6	6.4 ± 0.6	6.3 ± 0.9	6.5 ± 0.7	6.5 ± 0.7	6.6 ± 0.6	6.6 ± 0.6	6.8 ± 0.5	6.8 ± 0.4	6.8 ± 0.5	6.8 ± 0.5	6.6 ± 0.7	6.7 ± 0.6	-	-
Patient guidance	6.7 ± 0.6	6.6 ± 0.5	6.5 ± 0.7	6.6 ± 0.6	6.7 ± 0.6	6.7 ± 0.7	6.7 ± 0.6	6.8 ± 0.5	6.8 ± 0.4	6.8 ± 0.4	6.8 ± 0.4	6.7 ± 0.7	6.8 ± 0.5	-	-
**Overall didactic competencies**	6.6 ± 0.6	6.5 ± 0.5	6.2 ± 0.8	6.4 ± 0.5	6.6 ± 0.5	6.6 ± 0.5	6.6 ± 0.5	6.8 ± 0.5	6.6 ± 0.6	6.7 ± 0.5	6.8 ± 0.4	6.6 ± 0.6	6.7 ± 0.4	6.3 ± 1.0	6.5 ± 0.8
General didactic competence	6.6 ± 0.7	6.4 ± 0.6	6.2 ± 1.1	6.5 ± 0.7	6.6 ± 0.6	6.7 ± 0.6	6.6 ± 0.6	6.8 ± 0.5	6.7 ± 0.6	6.8 ± 0.4	6.8 ± 0.5	6.6 ± 0.8	6.7 ± 0.6	-	-
Communication	6.7 ± 0.7	6.6 ± 0.7	6.2 ± 1.2	6.5 ± 0.7	6.6 ± 0.7	6.7 ± 0.6	6.6 ± 0.7	6.8 ± 0.5	6.8 ± 0.7	6.9 ± 0.4	6.8 ± 0.5	6.6 ± 0.8	6.7 ± 0.6	-	-
Use of learning materials during course lesson	6.5 ± 0.8	6.5 ± 0.8	6.2 ± 1.0	6.3 ± 0.7	6.5 ± 0.8	6.5 ± 0.8	6.4 ± 0.8	6.7 ± 0.7	6.5 ± 0.9	6.6 ± 0.7	6.7 ± 0.6	6.6 ± 0.8	6.7 ± 0.5	-	-
Presentation	6.4 ± 0.8	6.1 ± 0.8	6.0 ± 1.0	6.2 ± 0.8	6.3 ± 0.8	6.4 ± 0.9	6.4 ± 0.8	6.7 ± 0.6	6.2 ± 1.1	6.4 ± 1.0	6.6 ± 0.7	6.5 ± 0.9	6.6 ± 0.6	-	-
Handling comments and questions	6.7 ± 0.7	6.6 ± 0.7	6.4 ± 1.0	6.6 ± 0.7	6.6 ± 0.7	6.6 ± 0.6	6.6 ± 0.7	6.8 ± 0.5	6.7 ± 0.7	6.8 ± 0.5	6.9 ± 0.4	6.7 ± 0.8	6.7 ± 0.5	-	-
Answering questions	6.7 ± 0.6	6.6 ± 0.6	6.4 ± 0.9	6.5 ± 0.7	6.6 ± 0.7	6.6 ± 0.6	6.6 ± 0.6	6.8 ± 0.5	6.7 ± 0.7	6.8 ± 0.5	6.8 ± 0.4	6.7 ± 0.6	6.7 ± 0.6	-	-
