# Supplementary material for: Quality assessment of a training program for undergraduate sonography peer tutors: paving the future way for peer-assisted learning in medical ultrasound education
Source: Front Med (Lausanne). 2025 Mar 3;12:1492596. doi: 10.3389/fmed.2025.1492596 (PMC11911324; doi:10.3389/fmed.2025.1492596)
Supplement: Supplementary file 6 [file Data_Sheet_6.pdf]

Supplement 6: Baseline of tutors from the semester 12-14

Item	Overall	Semester 12	Semester 13	Semester 14	p-value
Number of tutors	92	26	35	31	
Age (years)		25.4 ± 4.6	25.2 ± 3.3	24.8 ± 3.3	0.8
sex; n (%)<br>female (f)<br>male (m)	M = 31 (34%)<br>W = 61 (66%)	M = 8<br>W = 18	M = 13<br>W = 22	M = 10<br>W = 21	0.9
Number of courses held (n)	4.1 ± 5	4.1 ± 4.9	3.8 ± 4.5	4.4 ± 5.7	0.9
Number of independent sonographies (n)	172.1 ± 232.7	207.1 ± 399.5	137.5 ± 110.8	183.2 ± 139.3	0.5
Start of training (semester)					0.6
2. Preclinical	28 (30%)	7 (27%)	10 (29%)	11 (35%)	
3. Preclinical	18 (20%)	7 (27%)	6 (17%)	5 (16%)	
4. Preclinical	6 (7%)	3 (12%)	1 (3%)	2 (6%)	
1. Clinical (5th semester)	20 (22%)	6 (23%)	6 (17%)	8 (26%)	
2. Clinical (6th semester)	18 (20%)	3 (12%)	11 (31%)	4 (13%)	
3. Clinical (7th semester)	2 (2%)	0	1 (3%)	1 (3%)	
Current semester					0.1
4. Preclinical	11 (12%)	4 (19%)	1 (3%)	5 (16%)	
1. Clinical (5th semester)	15 (16%)	5 (19%)	7 (20%)	3 (10%)	
2. Clinical (6th semester)	20 (22%)	3 (12%)	12 (34%)	5 (16%)	
3. Clinical (7th semester)	14 (15%)	4 (15%)	2 (6%)	8 (26%)	
4. Clinical (8th semester)	11 (12%)	5 (19%)	3 (9%)	3 (10%)	
5. Clinical (9th semester)	15 (16%)	3 (12%)	8 (23%)	4 (13%)	
6. Clinical (10th semester)	5 (5%)	0	2 (6%)	3 (10%)	
7. Clinical (11th semester)	1 (1%)	1 (4%)	0	0	
