# Supplementary material for: Quality assessment of a training program for undergraduate sonography peer tutors: paving the future way for peer-assisted learning in medical ultrasound education
Source: Front Med (Lausanne). 2025 Mar 3;12:1492596. doi: 10.3389/fmed.2025.1492596 (PMC11911324; doi:10.3389/fmed.2025.1492596)
Supplement: Supplementary file 7 [file Data_Sheet_7.pdf]

Supplement 7: Tutor self-evaluation results per category across all semesters, and by semester

**Item**<br>(Likert scale 1=very low, 7=very high)	**Overall**	**Semester 12**	**Semester 13**	**Semester 14**	**p-value**
**Overall satisfaction**	5.1 ± 0.7	5.2 ± 0.8	4.9 ± 0.7	5.3 ± 0.7	0.1
**Working with the teaching material overall**	4.5 ± 0.9	4.2 ± 0.9	4.4 ± 0.9	4.9 ± 0.7	0.01
**Didactic competence overall**	5.3 ± 0.8	5.4 ± 0.6	5.1 ± 1.0	5.6 ± 0.6	0.1
**Specific ultrasound competence overall**	5.7 ± 0.6	5.7 ± 0.6	5.7 ± 0.6	5.8 ± 0.7	1.0
**Didactic and specific ultrasound competence overall**	5.5 ± 0.6	5.6 ± 0.6	5.4 ± 0.7	5.7 ± 0.6	0.3
**Social competence overall**	5.8 ± 0.6	5.7 ± 0.6	5.7 ± 0.7	5.9 ± 0.6	0.6
**Overall motivation**	5.8 ± 0.7	5.9 ± 0.7	5.8 ± 0.7	5.8 ± 0.8	0.9
