# Supplementary material for: Quality assessment of a training program for undergraduate sonography peer tutors: paving the future way for peer-assisted learning in medical ultrasound education
Source: Front Med (Lausanne). 2025 Mar 3;12:1492596. doi: 10.3389/fmed.2025.1492596 (PMC11911324; doi:10.3389/fmed.2025.1492596)
Supplement: Supplementary file 8 [file Data_Sheet_8.pdf]

Supplement 8: Theory test results in semesters 10 and 11

Item	Overall	Semester 10	Semester 11	p-value
**Number of tutors**	44	26	18	
**Age**	24.5 ± 3.5	25.1 ± 2.4	24.1 ± 4.1	0.4
**Sex**	M = 10 W = 34	M = 4<br>W = 14	M = 6<br>W = 20	1
**Current semester**	5.5 ± 1.7	5.8 ± 1.3	5.2 ± 2.0	0.3
**Total theory test score**<br>(max. 90 points)	67.2 ± 7.1	69.7 ± 5.7	65.5 ± 7.5	0.04
**Basics**<br>(max. 14 points)	13.0 ± 1.5	13.3 ± 1.1	12.7 ± 1.8	0.2
**Normal findings**<br>(max. 48 points)	34.8 ± 3.9	36.3 ± 3.3	33.9 ± 4.0	0.04
**Pathologies**<br>(max. 28 points)	19.4 ± 3.3	20.1 ± 2.7	18.9 ± 3.7	0.23
