# Supplementary material for: Quality assessment of a training program for undergraduate sonography peer tutors: paving the future way for peer-assisted learning in medical ultrasound education
Source: Front Med (Lausanne). 2025 Mar 3;12:1492596. doi: 10.3389/fmed.2025.1492596 (PMC11911324; doi:10.3389/fmed.2025.1492596)
Supplement: Supplementary file 9 [file Data_Sheet_9.pdf]

**Supplement 9:** Practical examination results between Semesters 11 and 13

| Item | Overall | Semester 11 | Semester 12 | Semester 13 | p-value |
|---|---|---|---|---|---|
| **Number of tutors** | 147 | 52 | 35 | 60 | |
| **total score** (max. 49 points) | 41.9 ± 5.1 | 40.2 ± 5.4 | 42.5 ± 3.8 | 43.1 ± 5.3 | 0.009 |
| **Patient guidance, communication and indication** (max. 10 points) | 9.1 ± 1.5 | 8.5 ± 1.8 | 9.4 ± 0.9 | 9.4 ± 1.2 | 0.004 |
| • Establishing a relationship (max. 2 points) | 1.9 ± 0.3 | 1.8 ± 0.5 | 2.0 ± 0.0 | 2.0 ± 0.1 | 0.004 |
| • Patient preparation (max. 2 points) | 1.9 ± 0.4 | 1.9 ± 0.5 | 1.9 ± 0.4 | 1.9 ± 0.3 | 0.5 |
| • Communication (max. 2 points) | 1.9 ± 0.4 | 1.8 ± 0.4 | 2.0 ± 0.2 | 1.9 ± 0.4 | 0.2 |
| • Breathing command (max. 2 points) | 1.8 ± 0.6 | 1.8 ± 0.4 | 1.7 ± 0.6 | 1.7 ± 0.6 | 0.8 |
| • Naming the indication (max. 2 points) | 1.8 ± 0.4 | 1.7 ± 0.5 | 1.8 ± 0.4 | 1.9 ± 0.3 | 0.03 |
| **Transducer handling and image optimization** (max. 8 points) | 7.3 ± 1.0 | 7.2 ± 1.2 | 7.4 ± 0.7 | 7.5 ± 1.0 | 0.2 |
| • Orientation (max. 2 points) | 1.9 ± 0.4 | 2.0 ± 0.0 | 1.8 ± 0.5 | 1.9 ± 0.4 | 0.03 |
| • Transducer positioning (max. 2 points) | 1.8 ± 0.5 | 1.8 ± 0.4 | 1.8 ± 0.5 | 1.9 ± 0.4 | 0.1 |
| • Transducer handling (max. 2 points) | 1.9 ± 0.4 | 1.9 ± 0.3 | 1.8 ± 0.5 | 1.9 ± 0.4 | 0.5 |
| • Image optimization (max. 2 points) | 1.7 ± 0.5 | 1.8 ± 0.4 | 1.7 ± 0.6 | 1.7 ± 0.6 | 0.9 |
| **Examination procedure** (max. 8 points) | 6.7 ± 1.7 | 6.4 ± 1.8 | 6.7 ± 1.7 | 6.9 ± 1.7 | 0.3 |
| **Image explanation** (max. 4 points) | 3.8 ± 0.7 | 3.7 ± 1.0 | 3.8 ± 0.5 | 3.9 ± 0.5 | 0.2 |
| **Measurement/advanced examination** (max. 6 points) | 5.1 ± 1.5 | 5.1 ± 1.4 | 5.3 ± 1.2 | 4.8 ± 1.6 | 0.3 |

| | | | | | |
|---|---|---|---|---|---|
| **Image documentation** (max. 1 point) | 0.5 ± 0.5 | 0.2 ± 0.4 | 0.4 ± 0.5 | 0.7 ± 0.5 | <0.001 |
| **Pathology assessment + description of further procedure** (max. 4 points) | 2.8 ± 1.2 | 2.4 ± 0.8 | 2.7 ± 1.3 | 3.2 ± 1.2 | 0.00011 |
| **Overall impression** (max. 8 points) | 6.7 ± 1.0 | 6.7 ± 1.0 | 6.7 ± 0.8 | 6.8 ± 1.2 | 0.4 |
