# Supplementary material for: Quality assessment of a training program for undergraduate sonography peer tutors: paving the future way for peer-assisted learning in medical ultrasound education
Source: Front Med (Lausanne). 2025 Mar 3;12:1492596. doi: 10.3389/fmed.2025.1492596 (PMC11911324; doi:10.3389/fmed.2025.1492596)
Supplement: Supplementary file 10 [file Data_Sheet_10.pdf]

Supplement 10: Results of the assessments comparison per semester and overall (across all semesters)

**Item**			**p-value**
**Semesters 12–14**			
	**Peer evaluation**	**Tutor self-assessment**	
Didactic competencies	93.2 ± 11.4	79.9 ± 12.3	< 0.001
Specific ultrasound competencies	92.8 ± 12.4	83.4 ± 9.1	< 0.001
Competence in total	83.9 ± 9.6	93 ± 11.3	< 0.001
**Semesters 12 and 13**			
	**Tutor self-assessment**	**Practical test results**	
Specific ultrasound competencies	83.3 ± 8.8	87.5 ± 9.8	0.007
Didactic competencies	86.1 ± 15.9	87.5 ± 9.8	0.5
**Semesters 10 and 11**			
	**Peer evaluation**	**Theory test results**	
Specific ultrasound competencies	95.0 ± 6.6	86.2 ± 9.1	< 0.001
Didactic competencies	95.5 ± 7.5	86.2 ± 9.1	< 0.001
Competence	95.2 ± 6.3	86.16 ± 9.1	< 0.001
**Semesters 11–13**			
	**Peer-evaluation**	**Practical test results**	
Specific ultrasound competencies	93.2 ± 11.3	85.6 ± 10.5	< 0.001
Didactic competencies	93.6 ± 10.7	85.6 ± 10.5	< 0.001
Competence in total	93.4 ± 10.4	85.6 ± 10.5	< 0.001
**Semester 11**			
	**Theory test**	**Practical test**	
Test results	85.0 ± 9.8	82.1 ± 11.0	0.3
**Across all semesters**			
	**Peer evaluation**	**Tutor self-assessment**	
Didactic competencies	93.8 ± 9.2	79.9 ± 12.3	< 0.001
Specific ultrasound competencies	94.0 ± 9.0	83.4 ± 9.1	< 0.001
Competence in total	93.9 ± 8.4	83.9 ± 9.6	< 0.001
	**Tutor self-assessment**	**Theory test results**	
Competence in total	83.4 ± 9.1	86.2 ± 9.1	0.1
	**Tutor assessment**	**Practical test results**	
Competence in total	83.4 ± 9.1	85.6 ± 10.5	0.1
	**Peer evaluation**	**Theory test results**	

Specific ultrasound competencies	94.0 ± 9.04	86.2 ± 9.1	< 0.001
Didactic competencies	93.8 ± 9.2	86.2 ± 9.1	< 0.001
Competence	93.9 ± 8.4	86.2 ± 9.1	< 0.001
	**Peer evaluation**	**Practical test results**	
Thematic competencies	94.0 ± 9.0	85.6 ± 10.5	< 0.001
Didactic competencies	93.6 ± 10.7	85.6 ± 10.5	< 0.001
Competence in total	93.9 ± 8.4	85.6 ± 10.5	< 0.001
	**Theory test**	**Practical test**	
Test results	86.2 ± 9.1	85.6 ± 10.5	0.8
